# Lack of Vacuolar H^+^ -Pyrophosphatase and Cytosolic Pyrophosphatases Causes Fatal Developmental Defects in *Arabidopsis thaliana*

**DOI:** 10.3389/fpls.2020.00655

**Published:** 2020-05-26

**Authors:** Mayu Fukuda, Marika Mieda, Ryosuke Sato, Satoru Kinoshita, Takaaki Tomoyama, Ali Ferjani, Masayoshi Maeshima, Shoji Segami

**Affiliations:** ^1^Laboratory of Cell Dynamics, Graduate School of Bioagricultural Sciences, Nagoya University, Nagoya, Japan; ^2^Department of Biology, Tokyo Gakugei University, Tokyo, Japan; ^3^Graduate School of Bioscience and Biotechnology, Chubu University, Kasugai, Japan; ^4^National Institute for Basic Biology, Okazaki, Japan

**Keywords:** *Arabidopsis thaliana*, cell wall, leaf atrophy, nitrogen nutrient, pyrophosphate, pyrophosphatase

## Abstract

The cytosolic level of inorganic pyrophosphate (PPi) is finely regulated, with PPi hydrolyzed primarily by the vacuolar H^+^-pyrophosphatase (H^+^-PPase, VHP1/FUGU5/AVP1) and secondarily by five cytosolic soluble pyrophosphatases (sPPases; PPa1–PPa5) in *Arabidopsis thaliana*. Loss-of-function mutants of H^+^-PPase (*fugu5*s) have been reported to show atrophic phenotypes in their rosette leaves when nitrate is the sole nitrogen source in the culture medium. For this phenotype, two questions remain unanswered: why does atrophy depend on physical contact between shoots and the medium, and how does ammonium prevent such atrophy. To understand the mechanism driving this phenotype, we analyzed the growth and phenotypes of mutants on ammonium-free medium in detail. *fugu5-1* showed cuticle defects, cell swelling, reduced β-glucan levels, and vein malformation in the leaves, suggesting cell wall weakening and cell lethality. Based on the observation in the double mutants *fugu5-1 ppa1* and *fugu5-1 ppa4* of more severe atrophy compared to *fugu5-1*, the nitrogen-dependent phenotype might be linked to PPi metabolism. To elucidate the role of ammonium in this process, we examined the fluctuations of sPPase mRNA levels and the possibility of alternative PPi-removing factors, such as other types of pyrophosphatase. First, we found that both the protein and mRNA levels of sPPases were unaffected by the nitrogen source. Second, to assess the influence of other PPi-removing factors, we examined the phenotypes of triple knockout mutants of H^+^-PPase and two sPPases on ammonium-containing medium. Both *fugu5 ppa1 ppa2* and *fugu5 ppa1 ppa4* had nearly lethal embryonic phenotypes, with the survivors showing striking dwarfism and abnormal morphology. Moreover, *fugu5 ppa1^+/–^ ppa4* showed severe atrophy at the leaf margins. The other triple mutants, *fugu5 ppa1 ppa5* and *fugu5 ppa2 ppa4*, exhibited death of root hairs and were nearly sterile due to deformed pistils, respectively, even when grown on standard medium. Together, these results suggest that H^+^-PPase and sPPases act in concert to maintain PPi homeostasis, that the existence of other PPi removers is unlikely, and that ammonium may suppress the production of PPi during nitrogen metabolism rather than stimulating PPi hydrolysis.

## Introduction

Vacuolar H^+^-translocating inorganic pyrophosphatase (H^+^-PPase; gene, VHP1) has two physiological roles: hydrolysis of PPi in the cytosol and active translocation of protons into plant vacuoles. PPi is generated as a byproduct of the synthesis of macromolecules, such as DNA, RNA, proteins, and polysaccharides (Stitt, 1998; Maeshima, 2000; Heinonen, 2001). Excessive accumulation of PPi in the cytosol suppresses macromolecule biosynthesis based on the law of mass action. On the other hand, several bi-directional enzymes involved in glycolysis, including pyrophosphate-dependent phosphofructokinase (PFP), UGPase, and pyruvate phosphate dikinase (PPDK) can both utilize and produce PPi (Hajirezaei et al., 1993; Heinonen, 2001; Park et al., 2010; Chastain et al., 2011). Thus, sufficient PPi is also essential for metabolic activities in plant cells. In addition to scavenging PPi, H^+^-PPase acts as a proton pump along with vacuolar H^+^-ATPase to maintain acidic pH within the vacuolar lumen, which occupies the largest volume within plant cells (Maeshima and Yoshida, 1989; Nakanishi et al., 2003). In most young tissues of plants, H^+^-PPase accounts for 10% of vacuolar membrane proteins by weight (Maeshima, 2001). Therefore, loss of H^+^-PPase activity is expected to markedly suppress plant growth. Surprisingly, the loss of H^+^-PPase function had a relatively mild phenotypic effects. A T-DNA insertion H^+^-PPase knockout mutant *vhp1-1* of *Arabidopsis thaliana* (*A. thaliana*) and amino acid exchange and deletion mutants of H^+^-PPase (*fugu5*s) showed abnormal cotyledon shape, with fewer and larger cells, when seedlings were grown in the absence of sucrose (Ferjani et al., 2011), as well as a mild suppression of plant growth (Asaoka et al., 2016), and delayed stomatal closure (Asaoka et al., 2019). Very recently, excess PPi has been reported to limit cotyledon pavement cell morphogenesis and to alter cotyledon flatness (Gunji et al., 2020). On the contrary, V-ATPase knockout mutant *vha-a2 vha-a3* showed severer growth defect and higher vacuolar pH than *fugu5*, suggesting that V-ATPase is the primary vacuolar proton pump (Krebs et al., 2010; Kriegel et al., 2015). Although another H^+^-PPase knockout mutant allele *avp1-1* (Li et al., 2005) displayed severe auxin-related growth defects, Kriegel et al. (2015) unambiguously demonstrated that *avp1-1* growth defects are due to a secondary T-DNA insertion in ARF-GEF GNOM gene, which is essential for PIN cycling. Few other studies suggested that H^+^-PPase can act as PPi synthase, providing PPi to sucrose oxidation pathway to energize sucrose loading into phloem (Pizzio et al., 2015; Khadilkar et al., 2016; Scholz-Starke et al., 2019).

The oblong shape of *fugu5* cotyledons recovered upon the addition of sucrose to the growth medium, as this phenotype was triggered by lowered sucrose production from seed storage lipids (Takahashi et al., 2017). Previous research into metabolite changes in *fugu5* seedlings using capillary electrophoresis time-of-flight mass spectrometry (CE-TOF-MS) and mathematical analysis revealed that UGPase is the major target of PPi’s inhibitory effect on gluconeogenesis, which ultimately leads to reduced sucrose production (Ferjani et al., 2018). In addition, double knockout mutants of H^+^-PPase and cytosolic soluble PPase (sPPase) exhibited marked changes in morphology and metabolites, including defect of cell wall components and excessive accumulation of starch, while sPPase quadruple mutants displayed a normal phenotype (Segami et al., 2018). Thus, H^+^-PPase has a greater impact on PPi homeostasis than that of sPPases.

Some non-plant species such as the purple photosynthetic bacterium *Rhodospirillum rubrum*, parasitic protozoa, *Streptomyces*, hyperthermophilic bacteria, and *Agrobacterium* possess H^+^-PPases (Baltscheffsky et al., 1999; Maeshima, 2000; Pérez-Castiñeira et al., 2001; Seufferheld et al., 2003; Hirono et al., 2007). In organisms without H^+^-PPase, such as *Escherichia coli* and *Saccharomyces cerevisiae*, loss of sPPase causes growth arrest and cell death due to hyperaccumulation of PPi (Chen et al., 1990; Serrano-Bueno et al., 2013) which were rescued by genetic complementation with H^+^-PPase (Pérez-Castiñeira et al., 2002). However, no lethal phenotype has been reported in plants to date. Other PPases such as PPi-dependent phosphofructokinase (Stitt, 1998; Amthor et al., 2019) may contribute to PPi hydrolysis in plants. In this study, we prepared multiple knockout mutants of H^+^-PPase and sPPase(s) to identify lethal conditions in plants and to evaluate the physiological contributions of four cytosolic soluble PPases (PPa1, PPa2, PPa4, and PPa5).

Recently, growth of *fugu5* and *vhp1* was found to be severely suppressed and cell death was observed at the basal region of the true leaves when grown on ammonium-free medium (Fukuda et al., 2016), which is commonly used for hydroponics. The phenotype was rescued either by addition of ammonium to the growth medium at more than 1 mM or genetic insertion of the yeast sPPase IPP1, indicating that excessive accumulation of PPi causes the observed phenotypic effects (Fukuda et al., 2016). Based on these observations, we explored the changes in the tissues of mutant lines grown under these specific conditions. In this study, we found that deletion of both H^+^-PPase and sPPase resulted in marked changes in the morphology and construction of cells and tissues, cell surface components, cell death rate, and development of plants, even in those grown on standard growth medium. These results reveal the importance of PPi homeostasis for nitrogen metabolism and amino acid biosynthesis as well as macromolecule and sucrose biosynthesis in plants. Here, we discuss the biochemical and physiological effects of excessive PPi on cell morphology and cell fate, with consideration of macromolecule biosynthesis and differences in nitrogen assimilation between roots and shoots.

## Materials and Methods

### Plant Materials and Growth Conditions

*A. thaliana* (accession Columbia-0; hereafter referred to as wild type, WT) seeds, which were provided by the RIKEN BioResource Center (Tsukuba, Japan), were surface-sterilized, placed in the dark at 4°C for 2 days and then sown on plates of 0.5× Murashige-Skoog (MS) medium containing 2.5 mM MES-KOH (pH 5.7), 1% (w/v) sucrose, and 0.6% gellan gum (0.5× MS plates) at 22°C under long-day conditions (light/dark regime of 16 h/8 h, cool-white lamps, 90 μmol/m^2^ s). In addition to WT, two loss-of-function mutant alleles of H^+^-PPase (Ferjani et al., 2007, 2011), also in the Columbia-0 background, were characterized under the same conditions. The PPa5-GFP which expresses cfSGFP2-tagged PPa5 under the control of its own promoter, the loss-of-function mutants of cytosolic soluble PPase (*ppa1*, *ppa2*, *ppa4*, and *ppa5*) and double mutants (*ppa1 ppa5*, *fugu5 ppa1*, *fugu5 ppa2*, *fugu5 ppa4*, and *fugu5 ppa5*) were prepared as described previously (Segami et al., 2018). Triple mutants (*fugu5 ppa1 ppa2*, *fugu5 ppa1 ppa4*, *fugu5 ppa1 ppa5*, and *fugu5 ppa2 ppa4*) were prepared through crossing of the corresponding mutant lines.

Molecular Genetics Research Laboratory (MGRL) culture medium and a modified form of it supplemented with NH_4_^+^ (MGRL^Am^) were prepared for examination of the effects of the NO_3_^–^ and NH_4_^+^ ions on plant growth. Basal MGRL medium for gel plates contained 1.5 mM NaH_2_PO_4_, 0.26 mM Na_2_HPO_4_, 1.5 mM MgSO_4_, 2.0 mM Ca(NO_3_)_2_, 3.0 mM KNO_3_, 12 μM Fe(III)-EDTA, 10 μM MnSO_4_, 30 μM H_3_BO_3_, 1.0 μM ZnSO_4_, 1.0 μM CuSO_4_, 24 nM (NH_4_)_6_Mo_7_O_24_, 130 nM CoCl_2_, 2% sucrose, and 0.4% gellan gum (Fujiwara et al., 1992; Naito et al., 1994). MGRL^Am^ medium contained 3.0 mM NH_4_Cl and 3.0 mM KCl instead of 3.0 mM KNO_3_, and thus contained 4 mM NO_3_^–^ and 3 mM NH_4_^+^ as the sole nitrogen sources. Medium pH of MGRL and MGRL^Am^ were adjusted to 5.8.

### Morphological Observations

Whole plants were observed and photographed using an EOS D60 (Canon) or EOS Kiss X7 digital camera (Canon) and a stereoscopic microscope (SZ61; Olympus) equipped with a CCD camera (DP50, Olympus or DSX500, Olympus). Embryos were cleared using Hoyer’s solution (Feng and Ma, 2017) and observed with a BX51 upright microscope (Olympus) equipped with a CCD camera (DP72, Olympus).

For observation of leaf veins, 10-, 15-, and 20-day-old leaves were fixed in a solution (ethanol:acetate = 3:1) at room temperature. The fixed specimens were washed in an ethanol series (70%, 50%, 30%, and 15%) and rinsed with ClearSee (10% xylitol, 15% sodium deoxycholate, and 25% urea) (Kurihara et al., 2015).

To observe the cross sections of rosette leaves, leaf samples including the leaf margin were cut to a size of 2 × 2 mm with a razor. Both ends of the leaves were sliced to allow the fixative solution to permeate well into the sample, and then the samples were immersed in a fixative solution (3% glutaraldehyde, and 50 mM Na-Pi, pH 7.0) and degassed thoroughly. The samples were embedded in 5% agar and sliced to 40 μm thickness with a microtome (VT 1200 s, Leica).

### Scanning Electron Microscopy (SEM)

Leaves were dissected from 10- and 20-DAG plants. They were mounted on a stub with adhesive carbon tape, and then transferred directly to a specimen chamber of the low-vacuum SEM (TM3030, Hitachi). Leaf epidermal cells were observed at 0–4°C, under low-vacuum conditions (30∼50 Pa).

### Confocal Laser Scanning Microscopy (CLSM)

Confocal laser scanning microscopy (CLSM) observations were conducted with an upright FV1000-D confocal laser scanning microscope (Olympus). For fluorescein diacetate (FDA) and propidium iodide (PI) staining, samples were soaked in dye solution containing 5 μg/ml FDA, 10 μg/ml PI, and 100 mM sorbitol. After 1 min, the samples were observed via CLSM. For calcofluor white staining, samples were fixed in 4% paraformaldehyde in phosphate-buffered saline (PBS) for 60 min under a vacuum at room temperature. The fixed tissues were washed twice for 1 min in PBS and cleared with ClearSee (Kurihara et al., 2015). The cleared samples were stained with 0.1% calcofluor white in ClearSee solution for 60 min and then washed with ClearSee solution for 30 min. The stained samples were observed via CLSM. UPLSAPO10X or UPLSAPO60XW (Olympus) was used as the objective lens. The excitation wavelength and transmission range for emission were 473 nm and 485 to 560 nm for FDA and green fluorescent protein (GFP), 559 nm and 617 to 717 nm for PI, and 405 nm and 425 to 475 nm for calcofluor white.

### Immunoblotting

Preparation of the soluble fraction from *A. thaliana* plants and immunoblotting were conducted as described previously (Segami et al., 2018). To detect PPa isozymes, a peptide-specific antibody for *A. thaliana* PPa1–PPa5 (C+MPMIDQGEKDDKII) was used.

### Toluidine Blue Staining

Whole plants grown on plate medium were stained with 0.1% toluidine blue for 2 min. After washing three times with distilled water, leaves were observed via a stereomicroscope (SZ61, Olympus).

### Image Analysis

Image analysis of leaf surface, leaf vein and area quantification was performed using an ImageJ Fiji (Schindelin et al., 2012). For quantification of the leaf areole density, in brief, the vessel patterns were traced manually, and subjected to “Analyze Particles” function to extract total areole area. Areole total area was divided by total leaf area. The obtained quotient was shown as an indicator of the leaf vein continuity.

For calculation of undulation index (UI), the cell perimeter and cell area of pavement cells were measured on SEM images (3∼7 leaves per one sample, more than 15 cells from one leaf). The complexity of the pavement cells was quantified by calculating the UI (Thomas et al., 2003) using the following equation (Kürschner, 1997):

$$U\,I=\frac{P}{2\,\pi \,\sqrt{A\,/\,\pi }}$$

where *UI* (dimensionless) is the undulation index, *P* (μm) is the cell perimeter, and *A* (μm^2^) is the cell area.

Quantification of leaf and seed area was performed using an ImageJ macro, as shown in Supplementary Figure 8. In brief, to select the desired area, Color Thresholder 2.0.0-rc-69/1.52p software was used. Then, the auto-generated macro code constructed by Color Thresholder was pasted into the macro at the indicated line.

## Results

### Morphological Phenotypes of Leaf of *fugu5*

**FIGURE 1:**
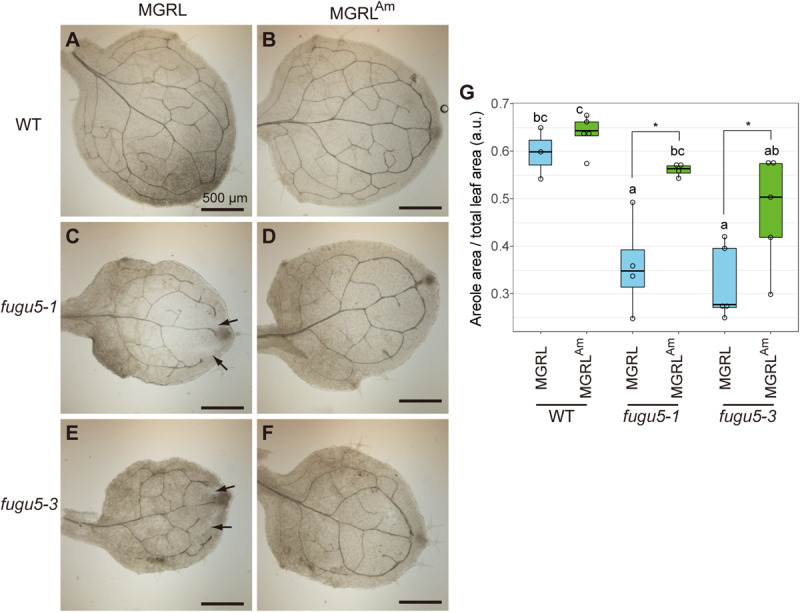
Phenotype of leaf veins of *fugu5* grown on MGRL culture medium. WT, *fugu5-1* and *fugu5-3* were grown on MGRL or MGRL^Am^ plates for 10 days. The first rosette leaves were collected and fixed for observation. Leaves of **(A)** WT, **(C)** *fugu5-1* and **(E)** *fugu5-3* grown on an MGRL plates and leaves of **(B)** WT, **(D)** *fugu5-1* and **(F)** *fugu5-3* grown on an MGRL^Am^ plates were photographed. Scale bars = 500 μm. Arrows indicate unconnected veins in *fugu5*. **(G)** Areole density. Different letters above each bar indicate statistically significant differences (*P* < 0.05, Tukey’s HSD test), and asterisks indicate statistically significant differences (*P < 0.05, Welch two sample *t*-test).

In *fugu5*, dead cells were observed in a highly proliferative region, namely the petiole-blade junction, of leaves grown on MGRL (Fukuda et al., 2016). To investigate further morphological changes in the other regions, we carefully observed the leaf veins, tissue construction of palisade mesophyll, and cell arrangement of the epidermis of true leaves. The first true leaves of 10-day-old plants grown on MGRL plates or plates with modified MGRL medium supplemented with 3 mM NH_4_Cl (MGRL^Am^) were fixed with a solution of ethanol and acetate, and then treated with ClearSee to visualize leaf veins. Normal networks of leaf veins were observed in WT grown on either MGRL or MGRL^Am^ plates (Figures 1A,B). In contrast, *fugu5-1* and *fugu5-3* leaves, particularly in the distal leaf region, did not form the normal network of veins when grown on MGRL plates (Figures 1C,E, arrows). This defect was clearly rescued when *fugu5-1* and *fugu5-3* was cultivated on MGRL^Am^ (Figures 1D,F). Statistical analysis of the images using the quotient of the areole area and total leaf area values confirm that *fugu5s* grown on MGRL had abnormal networks of leaf veins, in other word, networks with low leaf vein continuity (Figure 1G).

**FIGURE 2:**
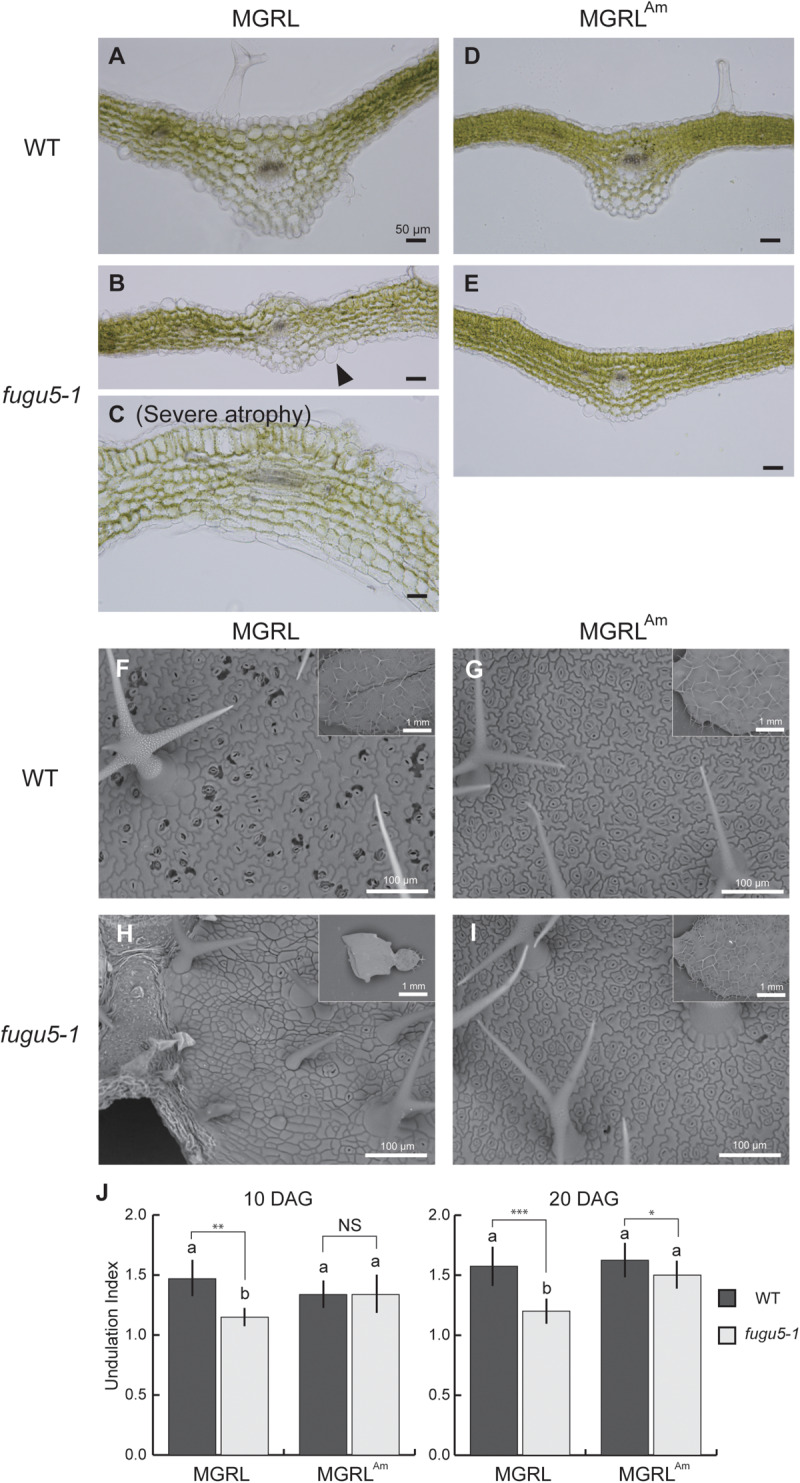
Abnormal epidermal and mesophyll cells in leaves of *fugu5* grown on MGRL culture medium. WT and *fugu5-1* were grown on an MGRL or MGRL^Am^ plates for 20 days. **(A–E)** Rosette leaves were fixed and sectioned for observation. Images of leaf cross sections from WT **(A)** and *fugu5-1* **(B,C)** grown on an MGRL plates, and from WT **(D)** and *fugu5-1* **(E)** grown on MGRL^Am^ plates were taken using a upright microscope. Arrowheads indicate the swollen cells in epidermis. **(F–I)** SEM images of leaf adaxial side. Leaves of **(F)** WT and **(H)** *fugu5-1* grown on an MGRL plates and leaves of **(G)** WT and **(I)** *fugu5-1* grown on an MGRL^Am^ plates observed. **(J)** UI of pavement cells. First leaves of 10-DAG plants and third or fourth leaves of 20-DAG plants were used for the observations above, except for severely atrophied *fugu5-1* grown on MGRL, in which the leaf stage was hardly distinguished. Different letters above each bar indicate statistically significant differences (*P* < 0.05, Tukey’s HSD test), and asterisks indicate statistically significant differences at *P < 0.05, **P < 0.01, ***P < 0.001 (Welch two sample *t*-test, *n* > 50). Error bars indicate SD (*n* > 50).

Next, we observed cross sections of true leaves to investigate tissue construction. Generally, leaf mesophyll tissue is composed of four to five layers of cells of similar size. In 20-day-old plants, leaves from WT grown on both MGRL and MGRL^Am^ and *fugu5-1* grown on MGRL^Am^ showed the normal arrangement of cells of regular size (Figures 2A,D,E). In *fugu5-1* leaves, the alignment of cells (cell layers) was irregular and cell size was variable (Figures 2B,C). Furthermore, several large cells were present in the pavement cells of *fugu5-1* grown on MGRL (Figure 2B, arrowhead). *fugu5-1 ppa1* and *fugu5-1 ppa4* plants grown on MGRL^Am^ showed severe phenotypic effects, including cell swelling and abnormal cell alignment (Supplementary Figure 1), suggesting that PPi accumulation caused cell swelling in leaves. Notably, *fugu5-1 ppa1* grown on MGRL showed cell death in the adaxial side of the leaf, while *fugu5-1 ppa4* showed cell death in the leaf margins (Supplementary Figure 1).

To detect morphological differences in pavement cells of 20-day-old leaves, SEM analysis was performed. Pavement cells usually exhibit puzzle-cell formation, but very recently Gunji et al. (2020) reported that the complexity of cotyledon pavement cells was reduced by PPi accumulation via inhibiting microtubule dynamics. On both MGRL and MGRL^Am^, WT leaves showed normal pavement cell structure (Figures 2F,G). However, *fugu5-1* grown on MGRL showed obviously simplified cells (Figure 2H), while *fugu5-1* grown on MGRL^Am^ showed puzzle-cell formation (Figure 2I), suggesting ammonium deficient conditions likely increased PPi level in pavement cells. Statistical analysis of UI, which indicates the degree of cell structure complexity, confirmed the above observations (Figure 2J).

To check whether nitrogen content was affected by *fugu5* mutation, ammonium and nitrate contents were analyzed in plant shoots. For ammonium content, there was no significant difference between the WT and *fugu5-1*, although shoots grown on MGRL^Am^ accumulated twice more ammonium than MGRL in 10-DAG plants (Supplementary Figure 2A). On the other hands, in 10-DAG plants, there was no large difference in nitrate content except a little decrease in the WT grown on MGRL^Am^ (Supplementary Figure 2C). In 20-DAG plants grown on MGRL^Am^, both the WT and *fugu5-1* consumed ammonium and nitrate (Supplementary Figures 2B,D). Together, these results imply that there were no large differences between WT and *fugu5-1* in nitrogen usage.

### Defects in the Cell Wall and Cuticle Layer of Mutant Leaf Epidermis

In roots, *fugu5-1 ppa1* showed cell swelling, which was likely caused by decreased cellulose and root tip burst due to hypotonic treatment (Segami et al., 2018). Previously, we reported that contact of leaves with the culture medium was closely related to the occurrence of leaf atrophy (Fukuda et al., 2016). Therefore, we estimated that cell death in *fugu5* leaves on MGRL is caused by extracellular abiotic stresses.

**FIGURE 3:**
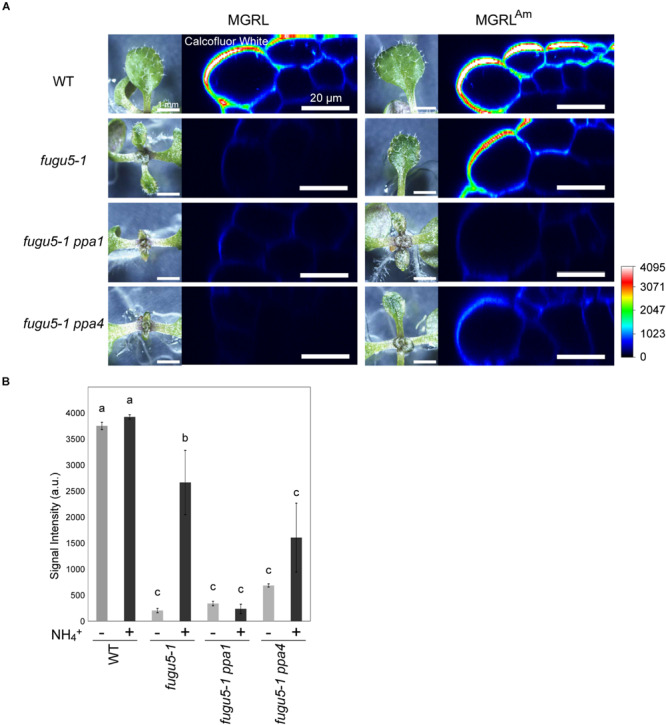
Lack of β-glucan in the cell wall of leaf epidermis of mutants grown on MGRL culture medium. **(A)** X-Z section of calcofluor white-stained leaves of WT, *fugu5*, *fugu5 ppa1*, and *fugu5 ppa4*. Plantlets grown on MGRL or MGRL^Am^ plates for 10 days were observed using stereomicroscopy (left panels) and CLSM (right panels). **(B)** Fluorescence intensity of calcofluor white signal at the rim of leaves. Seedlings grown on MGRL (–) or MGRL^Am^ (+) plates were compared. The fluorescence of the outermost cell wall of leaf epidermis was quantified and the average signal intensities are shown. Error bars indicate standard error (*n* = 3). Different letters above each bar indicate statistically significant differences (*P* < 0.05, Tukey’s HSD test).

To check for deficiencies of cell wall components, we stained 10-day-old leaves with calcofluor white, a fluorescent dye that binds to β-glucan (Anderson et al., 2010), and observed the epidermal cells at leaf margins with CLSM (Figure 3). *fugu5-1* grown on MGRL showed a strong decrease in the fluorescence signal and *fugu5-1* grown on MGRL^Am^ showed a mild decrease in signal intensity compared with WT grown on both MGRL and MGRL^Am^. The double mutants *fugu5-1 ppa1* and *fugu5-1 ppa4*, particularly the former, which exhibited a severe leaf phenotype (Supplementary Figure 1), also showed markedly low signals. These results suggest a relationship between the β-glucan content and mechanical strength of the cell wall.

**FIGURE 4:**
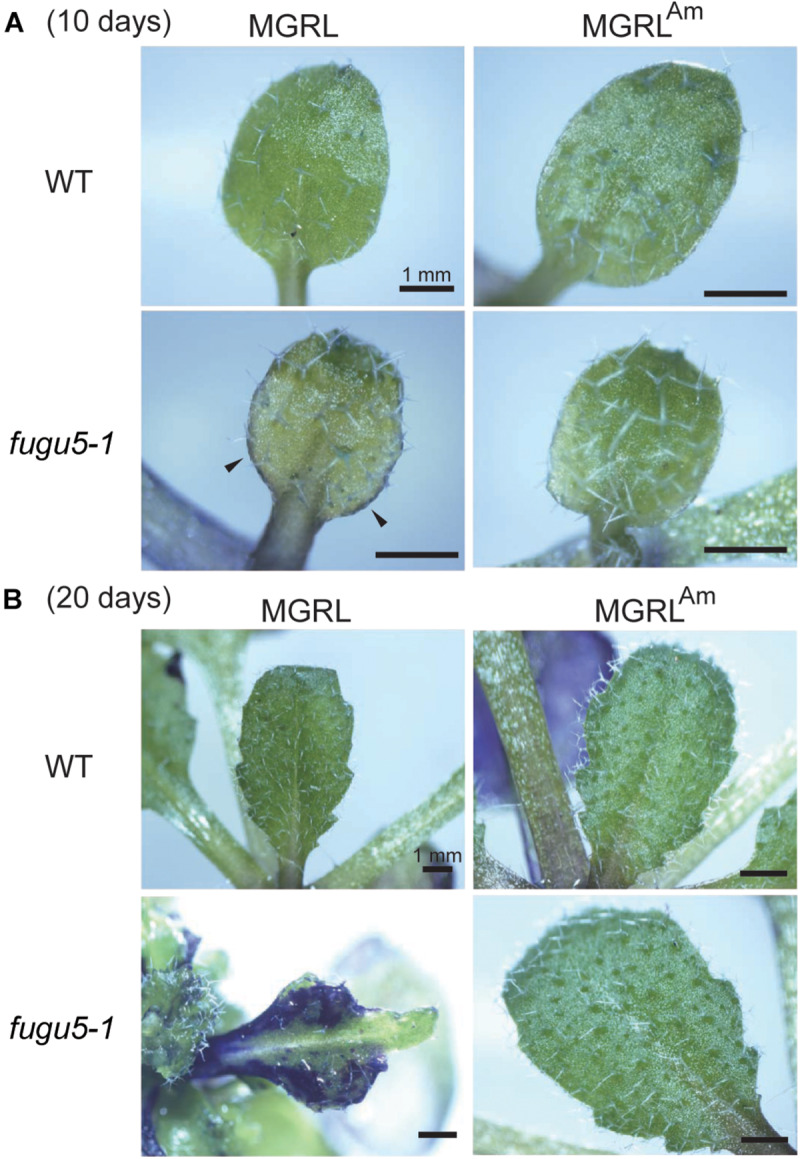
Partial lack of cuticle layer of *fugu5* grown on MGRL culture medium. Whole shoots grown on MGRL or MGRL^Am^ were treated with toluidine blue. **(A)** The first leaves of 10-DAG WT and *fugu5-1* grown on MGRL (left panel) or MGRL^Am^ plates (right panel) were observed with a stereomicroscope. **(B)** Rosette leaves of 20-day-old WT and *fugu5-1* on MGRL (left) or MGRL^Am^ plates (right) were observed. Regions stained dark blue lack a cuticle layer on the leaf surface.

Generally, the surface of plant shoot tissues is covered with a thin hydrophobic layer called the cuticle (Fich et al., 2016). The cuticle prevents water loss from the leaf surface and entry of water and solutes. The cuticle is a multilayered structure composed of waxes and related hydrocarbons deposited on the leaf epidermis. To examine the integrity of the cuticles of WT and *fugu5* leaves, fresh leaves were stained with toluidine blue, which is a water-soluble dye with high affinity for acidic components. If the leaf surface lacks a cuticle layer, the dye would stain the cells (Tanaka et al., 2004). WT leaves grown on both plate media did not stain (Figures 4A,B). In contrast, *fugu5-1* leaves grown on MGRL stained moderately (10-day-old seedling; Figure 4A) or strongly at the rim of basal region (20-day-old; Figure 4B), which coincides with the atrophic region. These results indicate that the epidermal cells of *fugu5-1* are unable to synthesize the components to generate adequate cuticles.

### Double Mutants Grown in Ammonium-Free Medium Exhibit Severe Growth Defects

**FIGURE 5:**
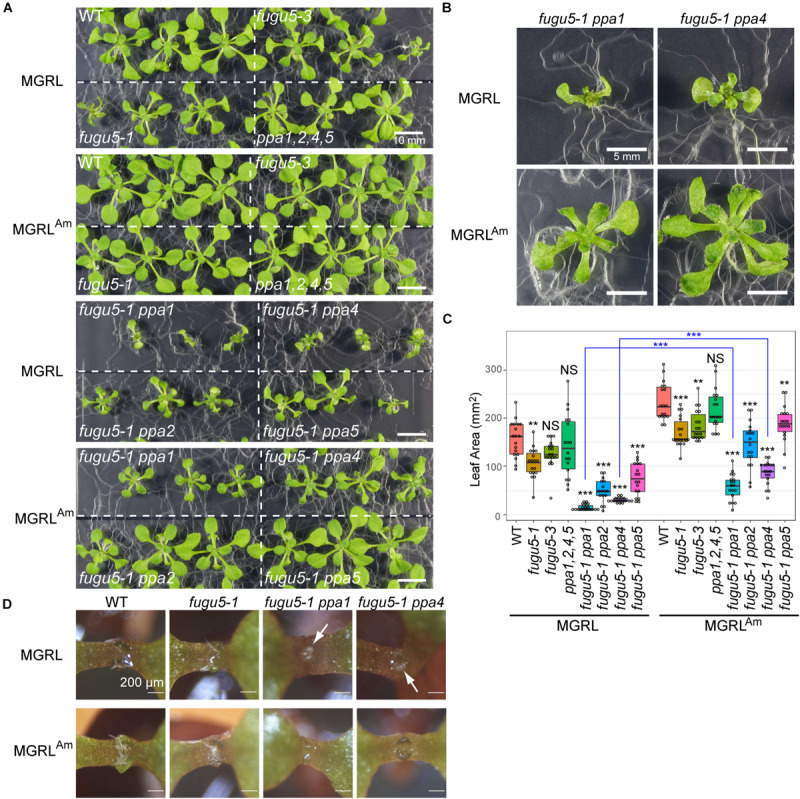
Effect of NH_4_^+^ in culture medium on growth. **(A)** Growth of WT, *fugu5-1*, *fugu5-3*, *ppa1,2,4,5*, *fugu5-1 ppa1*, and *fugu5-1 ppa4* seedlings for 17 days on MGRL (without NH_4_^+^) or MGRL^Am^ (3 mM NH_4_^+^) plates. **(B)** Magnified images of *fugu5-1 ppa1* and *fugu5-1 ppa4* grown on MGRL or MGRL^Am^. **(C)** Leaf area was calculated using photographs and represented with boxplots and dot plots. Asterisks above the boxplots indicate statistically significant differences of mean values compared with the WT of each medium condition (**P < 0.01, ***P < 0.001, Steel test), and blue asterisks between pair indicate statistically significant differences of variances (***P < 0.001, *F* test). **(D)** Seedlings were grown on MGRL or MGRL^Am^ plates for 6 days using plastic sheets to prevent contact between cotyledons and medium. Arrows indicate areas of chlorosis (small true leaves).

Leaf atrophy in *fugu5-1* depends on other factors, such as contact of leaves with the medium surface and agar concentration (i.e., medium hardiness) (Fukuda et al., 2016). Therefore, the observed phenotypes varied among independent experiments. Additionally, we found striking leaf atrophy in *fugu5-1 ppa1* and *fugu5-1 ppa4* plants grown on MGRL. These mutants are known to show a weak leaf atrophy phenotype when grown in half-strength MS, which contains 10 mM ammonium (Segami et al., 2018). All individuals of the double mutants failed to fully expand leaves when grown on MGRL (Figures 5A,B). This phenotype was stable, and the variance of leaf area for seedlings grown on MGRL was significantly lower than that on MGRL^Am^ for each line (*F* test; Figure 5C). Moreover, in 6-DAG seedlings grown with plastic sheets, which prevent direct contact of cotyledons with the growth medium, *fugu5-1 ppa1* and *fugu5-1 ppa4* grown on MGRL showed chlorosis in the emerging true leaves (Figure 5D, arrows). These results suggest that the phenotype exhibited by *fugu5-1 ppa1* and *fugu5-1 ppa4* grown on MGRL was independent of plant-medium contact and appeared at younger stage than that of *fugu5-1*.

### sPPase Level Was Not Affected by Supply of Ammonium Ion

A primary question of this study is how ammonium ions prevent atrophy of *fugu5* mutants. It has been shown that atrophy is triggered by excess PPi (Fukuda et al., 2016). Therefore, we postulated three possible effects of ammonium: (1) induction of sPPase expression; (2) induction of the expression of other pyrophosphatases; and/or (3) suppression of PPi generation in the mutants.

**FIGURE 6:**
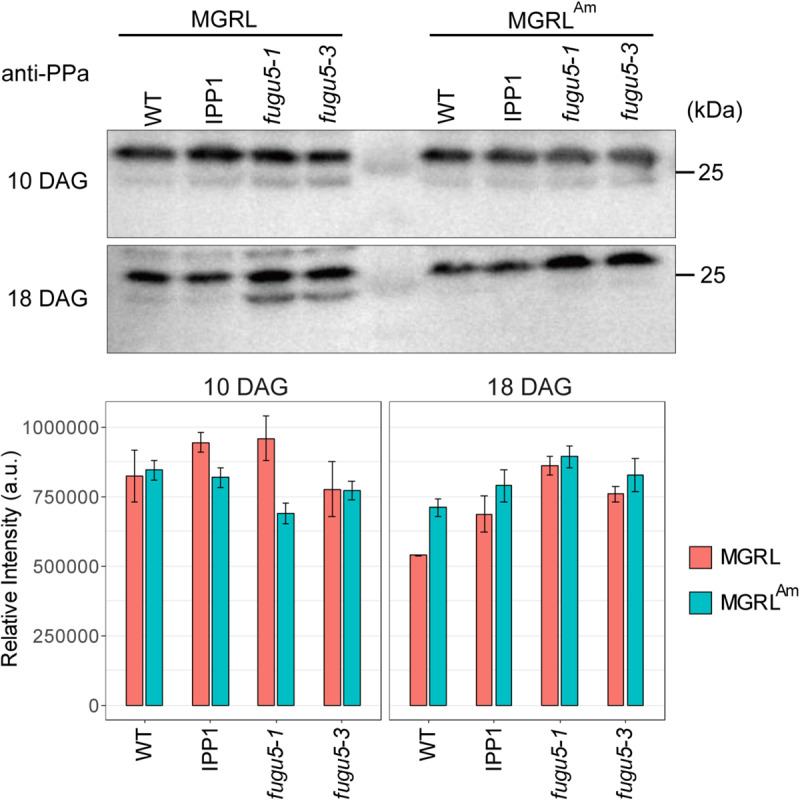
Immunoblot analysis of PPases in the soluble fractions from WT and mutants. **(A)** WT, a mutant expressing yeast cytosolic PPase (IPP1), *fugu5-1*, and *fugu5-3* were grown on MGRL or MGRL^Am^ plates for 10 (upper panel) or 18 days (lower panel). Soluble fractions were prepared and subjected to immunoblotting using anti-sPPase. sPPases were detected at 27 and 25 kDa. **(B)** Relative intensities of immunostained bands at 27 kDa are indicated with orange (MGRL) and blue boxes (MGRL^Am^).

To test the first possibility, we prepared soluble fractions from WT and mutants grown on MGRL and MGRL^Am^, and then performed immunoblotting using anti-sPPase, which collectively detects five sPPases, PPa1 to PPa5, to examine their protein level (Segami et al., 2018). There was no marked change in the level of sPPases between samples grown on the two types of media (Figure 6). In addition, we measured the mRNA levels of four sPPases and four PFPs (At1g12000, At1g20950, At1g76550, and At4g04040) and found no marked differences between MGRL and MGRL^Am^ conditions (Supplementary Figure 3 and Supplementary Table 1). These results suggest that ammonium does not induce the expression of sPPases or PFPs.

### Seed Viability Defects of *fugu5 ppa1 ppa2* and *fugu5 ppa1 ppa4*

To further validate the existence of other PPi-hydrolysis enzymes or PPi-removing factors, we prepared double and triple knockout mutants for various combinations of H^+^-PPase and sPPases. If these mutants are lethal, the existence of other functional PPi removal mechanisms is unlikely. All double mutants of H^+^-PPase and sPPase, including the most severe mutant *fugu5 ppa1*, showed normal fertility (data not shown). However, numerous shrunken seeds were observed in the triple heterozygous mutants *fugu5-1 ppa1 ppa2* and *fugu5-1 ppa1 ppa4* (Supplementary Figure 4A). It is worth to notice that non-shrunken seeds from *fugu5 ppa1^+/–^ ppa2* and *fugu5 ppa1^+/–^ ppa4* parental lines showed massive increase in size (Supplementary Figure 4B). The other line with shrunken seeds, *fugu5-1^+/–^ ppa1 ppa2*, showed less size increase, suggesting that nutrient surplus due to inhibited development of 25% of seeds did not cause the size increase. Interestingly, *fugu5-1* and sPPase quadruple mutant *ppa1*,*2*,*4*,*5* formed larger seeds. It appears again that increased PPi somehow affected seed storage oil metabolism, in agreement with the previous report by Meyer et al. (2012), in which seed specific overexpression of PPa1 and RNAi of PPa1 or PPa4 have been analyzed.

To test the fertility of the triple mutants, we tested the genotypes of heterozygous mutant progeny. Both *fugu5-1 ppa1 ppa2* and *fugu5-1 ppa1 ppa4* germinated from freshly prepared seeds, but not from old seeds that had been stored for at least 1 year (Tables 1, 2). The germination rates of *fugu5 ppa1 ppa2* and *fugu5 ppa1 ppa4* were considerably reduced. These results suggest that both *fugu5 ppa1 ppa2* and *fugu5 ppa1 ppa4* are nearly lethal and not tolerant of long-term storage.

**TABLE 1:** Segregation ratios of triple mutants by genotype.

			**Genotype of seedlings**			
**Genotype of parent**	**Storage^#^**	**Total**	**–/–**	**+/–**	**+/+**	**Ungerminated**
*fugu5-1^+/–^ ppa1 ppa2*	0 days	84	4	45	20	15
	4 years	41	0	19	7	15*
*fugu5-1 ppa1^+/–^ ppa*2	1 year	74	0	39	32	3*
*fugu5-1 ppa1 ppa2^+/–^*	1 year	82	0	45	23	14*
*fugu5-1 ppa1^+/–^ ppa4*	0 days	90	8	44	29	9
*fugu5-3^+/–^ ppa1 ppa4*	2 weeks	44	2	27	15	13

^#^Storage refers to the period from seed harvest to sowing. *Some shrunken seeds were removed during the harvest process. In other cases, seeds were directly harvested from individual fruits and sowed immediately.

**TABLE 2:** Segregation ratios of triple mutants by phenotype.

		**Seedling phenotype**				
**Genotype of parent**	**Storage**	**Total**	**Dwarf**	**Curled**	**Normal**	**Ungerminated**
*fugu5-1 ppa1^+/–^ ppa4*	1 year	89	0	57	24	8*
*fugu5-3^+/–^ ppa1 ppa4*	1 year	88	0	0	75	13*

Plants were classified by phenotype: Normal means no obvious phenotypic effects or marginal atrophy (*ppa1 ppa4* or *fugu5-1 ppa4-*like), Curled indicates strong leaf edge atrophy (*fugu5 ppa1^+/–^ ppa4-*like) and Dwarf represents severe growth defect (*fugu5 ppa1 ppa4-*like). See Figure 8B for morphological examples. *Some shrunken seeds were removed during the harvest process.

**FIGURE 7:**
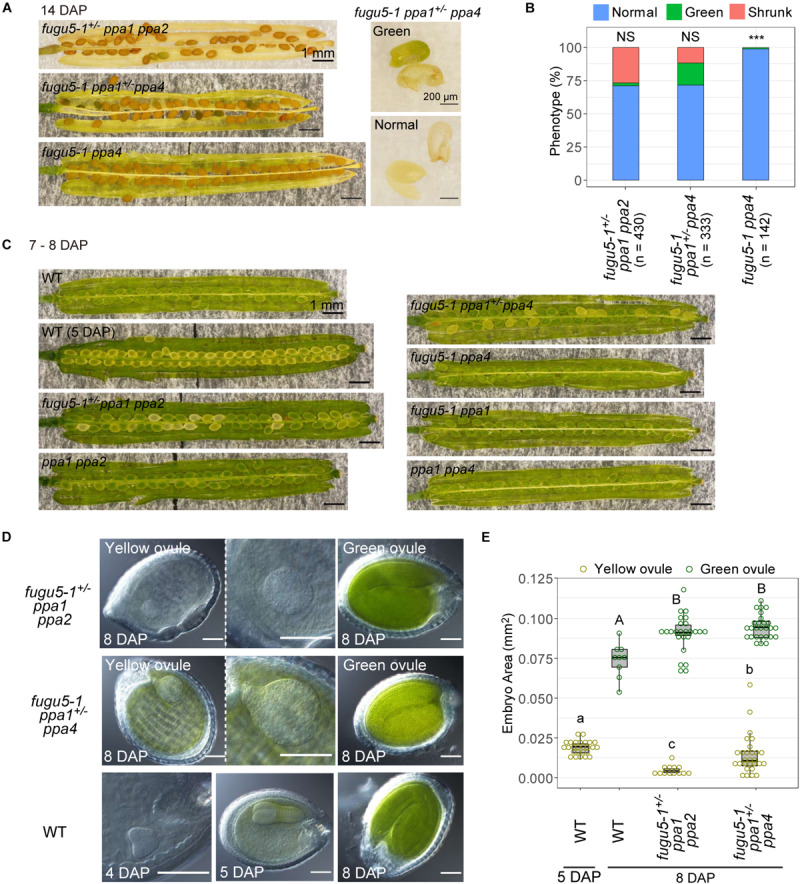
Defect in seed development of *fugu5 ppa1 ppa2* and *fugu5 ppa1 ppa4*. **(A)** 14-DAP fruits of *fugu5-1^+/–^ ppa1 ppa2, fugu5-1 ppa1^+/–^ ppa4* and *fugu5-1 ppa4* (left). A green embryo and its seed coat of *fugu5-1 ppa1^+/–^ ppa4* (right, green). The lower panel shows a normal embryo and its seed coat (normal). **(B)** Developmental defect of seeds in 14-DAP fruits of mutant lines. Ratios of normal (light blue), greenish (green), and shrunken seeds (orange) are shown for each line. Asterisks indicate significant differences in the proportion of normal seeds compared with 0.75 (Mendelian segregation) (*** *P* < 0.001, prop test). NS, not significant. **(C)** Images of fruits of WT and mutants at 7–8 DAP. **(D)** Ovules were treated with Hoyer’s solution to achieve organ transparency and then photographed. **(E)** Embryo sizes of WT and mutants were calculated as their area in photographic images and represented by boxplots and dot plots. Different letters above each bar indicate statistically significant differences (*P* < 0.01, Steel-Dwass test).

To determine when these mutations caused seed defects, we observed ovules and embryos. At 14 DAP, when the valves turned yellow, about 75% of seeds of both *fugu5-1^+/–^ ppa1 ppa2* and *fugu5-1 ppa1^+/–^ ppa4* showed normal brown coloration, while the remaining 25% of seeds appeared abnormal, either green or shrunken, indicating that the ratio of these defective seeds followed Mendelian segregation (Figures 7A,B). In *fugu5-1 ppa1^+/–^ ppa4*, green seeds contained green embryos with their cotyledons and hypocotyls fused together (Figure 7A). The genotypes of most green embryos were identified as *fugu5-1 ppa1 ppa4* homozygous (Supplementary Figure 5).

### Undeveloped Embryos in *fugu5 ppa1 ppa2* and *fugu5 ppa1 ppa4*

At 7–8 DAP, WT and all double mutants had green ovules, which is the normal color at this developmental stage (Figure 7C). The color of ovules varies during embryonic development, and WT at 5 DAP had a torpedo-stage embryo (Figure 7D) with yellow ovule coloration (Figure 7C). At 8 DAP, triple mutants *fugu5^+/–^ ppa1 ppa2* and *fugu5 ppa1^+/–^ ppa4* heterozygous, contained about 25% yellow ovules (Figure 7C). For further analysis of the embryos in these ovules, we observed transparent ovules cleared with Hoyer’s solution (Feng and Ma, 2017). Yellow ovules of *fugu5-1^+/–^ ppa1 ppa2* and *fugu5 ppa1^+/–^ ppa4* heterozygous had malformed spherical embryos, whereas the embryos of green ovules exhibited bent cotyledons. The size of yellow ovule embryos was larger than that of the heart stage of the WT at 4 DAP and smaller than that of the WT torpedo stage at 5 DAP (Figures 7D,E, yellow ovule). In particular, *fugu5 ppa1^+/–^ ppa4* had larger embryos than did *fugu5^+/–^ ppa1 ppa2* heterozygous. These observations indicate that the embryos were not simply delayed in development but also grew abnormally.

### Indispensability of H^+^-PPase and sPPases for Shoot Development

**FIGURE 8:**
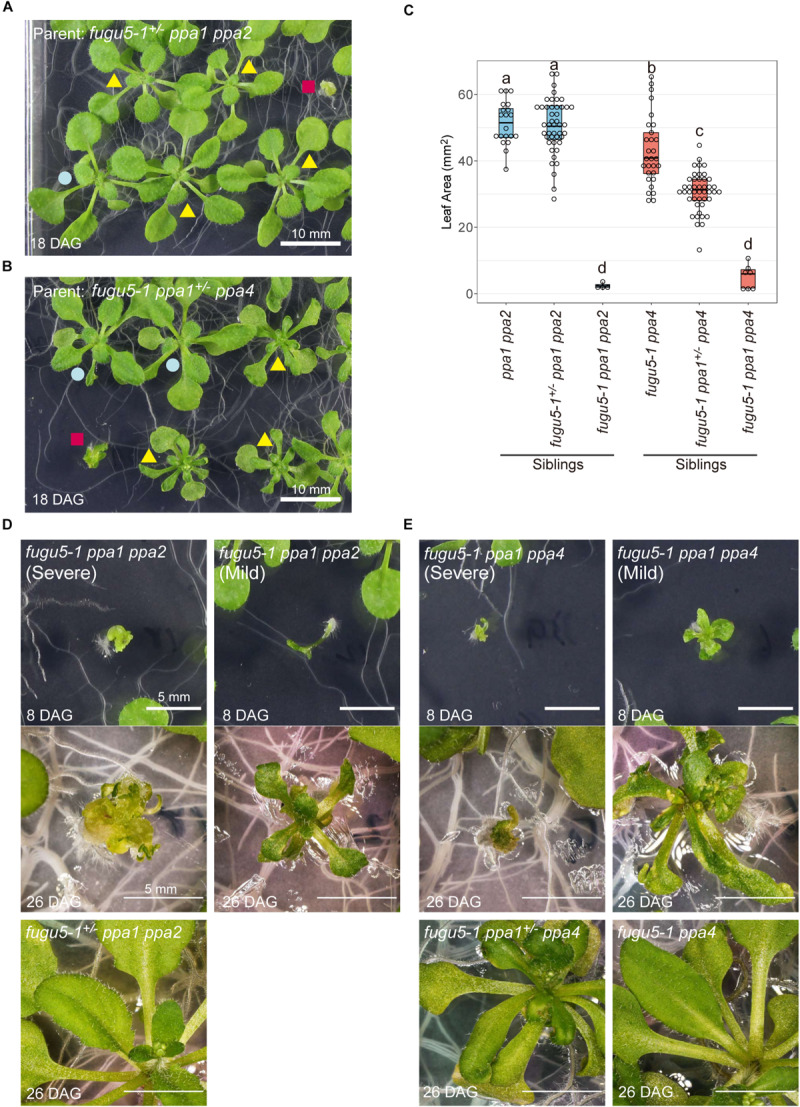
Defect in development of the triple mutants *fugu5-1 ppa1 ppa2* and *fugu5 ppa1 ppa4*. **(A)** 18-DAG plants germinated from *fugu5-1^+/–^ ppa1 ppa2* seeds. Red square indicates *fugu5-1 ppa1 ppa2*, yellow triangles indicate *fugu5-1^+/–^ ppa1 ppa2* and blue circle indicates *ppa1 ppa2*. **(B)** 18-DAG plants germinated from *fugu5-1 ppa1^+/–^ ppa4* seeds. Red square indicates *fugu5-1 ppa1 ppa4*, yellow triangles indicate *fugu5-1^+/–^ ppa1 ppa4* and blue circle indicates *ppa1 ppa4*. **(C)** Comparison of leaf area of 12-DAG plants. Different letters above each plot indicate statistically significant differences (*P* < 0.05, Tukey’s HSD test). **(D)** Phenotype of plant from *fugu5-1 ppa1 ppa2*. **(E)** Phenotype of *fugu5-1 ppa1 ppa4*. Plants were grown on half-strength MS plates with sucrose.

Both triple mutants *fugu5-1 ppa1 ppa2* and *fugu5-1 ppa1 ppa4* showed extremely reduced shoot growth (Figures 8A–C). On the other hand, the heterozygous mutant *fugu5-1^+/–^ ppa1 ppa2* showed normal morphology and no significant differences from *ppa1 ppa2* (Figures 8A,B), suggesting that a single copy of the VHP1 gene is sufficient to compensate for lack of PPa1 and PPa2. Another heterozygous mutant, *fugu5-1 ppa1*^+/–^ *ppa4*, showed decreased leaf area compared with *fugu5-1 ppa4* (Figure 8C) and a strong leaf edge curling phenotype, even when grown on MS plates, on which *fugu5* showed no atrophic symptoms (Figure 8B; yellow triangle). At 8-DAG, both *fugu5-1 ppa1 ppa2* and *fugu5-1 ppa1 ppa4* showed extremely abnormal shapes including apparent lack of cotyledons, short roots, and distorted leaves (Figures 8D,E). At 26-DAG, several individuals of *fugu5-1 ppa1 ppa2* and *fugu5-1 ppa1 ppa4* successfully developed leaves, but they remained very small compared with heterozygous or double mutant plants (Figures 8D,E; right and lower panels), and were especially damaged in *fugu5-1 ppa1 ppa4* (Figure 8E; right panel). In severe cases, individuals of *fugu5-1 ppa1 ppa2* and *fugu5-1 ppa1 ppa4* had no expanded leaves (Figures 8D,E; left panels). These results indicate that PPa1, PPa2, and PPa4 are active in the true leaves and contribute to PPi homeostasis in the *fugu5* background.

In MGRL medium, the double mutants *fugu5-1 ppa2* and *fugu5-1 ppa5* exhibited reduced leaf area but not severe atrophy (Figures 5A,C). In addition, *fugu5-1 ppa1*^+/–^ *ppa2* showed no leaf atrophy on MS medium (Supplementary Figure 6A), despite significantly reduced leaf area and root length (Supplementary Figures 6B–E), suggesting that PPa2 does not contribute to preventing leaf atrophy.

Another triple mutant *fugu5-3 ppa2 ppa4* had full viability but retarded cotyledon development compared with its heterozygous siblings *fugu5-3 ppa2^+/–^ ppa4* and *fugu5-3 ppa2 ppa4^+/–^* (Supplementary Figures 7A,B). Their cotyledon shape was distorted, and more than half of the plants formed single cotyledons. Six out of 26 seedlings of this triple mutant failed to form true leaves and their growth was prematurely arrested (Supplementary Figures 7A,C). These observations indicate that VHP1, PPa2, and PPa4 are essential for normal development of the cotyledons and shoot apical meristem at an early developmental stage, and that PPa1 alone can maintain PPase activity at a sufficient level for viability.

In addition, *fugu5-3 ppa2 ppa4* showed severe floral phenotypic changes, including enlarged and distorted pistils (Supplementary Figure 7D). Although few seeds were obtained from the mutant plant, hand pollination using WT pollen was not successful. In contrast, *fugu5-3 ppa2 ppa4* pollen succeeded in pollinating WT pistils (data not shown), suggesting that the pistil of *fugu5-3 ppa2 ppa4* is impaired. The petals and sepals of the mutant also showed morphological abnormalities (Supplementary Figure 7D).

### Root Hair Cell-Specific Necrosis in *fugu5 ppa1 ppa5*

**FIGURE 9:**
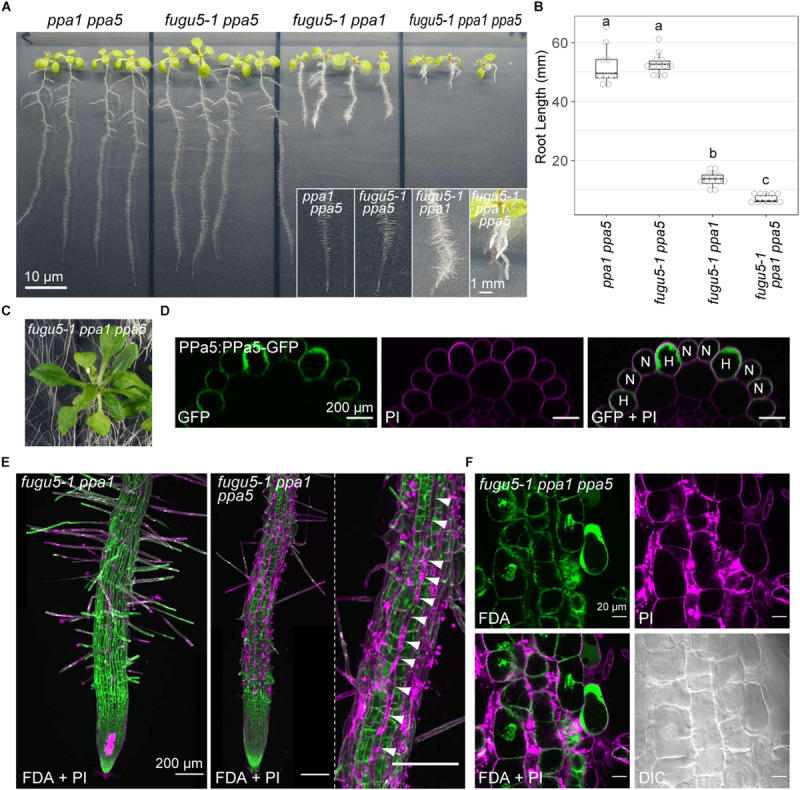
Severe phenotype of the triple mutant *fugu5-1 ppa1 ppa5*. **(A)** Loss-of-function mutants (*ppa1 ppa5*, *fugu5-1 ppa5*, *fugu5-1 ppa1*, and *fugu5-1 ppa1 ppa5*) were grown on half-strength MS plates with 1% sucrose for 9 days. Lower insets show enlarged images of roots. **(B)** Root lengths of the WT and mutants were measured and visualized as boxplots and circles (*n* = 3). Different letters above each plot indicate statistically significant differences (*P* < 0.05, Tukey’s HSD test). **(C)** Aboveground organs of 30-DAG *fugu5-1 ppa1 ppa5* plants. **(D)** X-Z images constructed from Z-stack images of PPa5-GFP stained with PI. Left, GFP image; middle, PI image; and right, a merged image of GFP and PI. H, root hair cell; N, non-root hair cell. **(E)** Live/dead-cell staining of roots collected from the seedlings shown in **(A)**. Roots were stained with fluorescein diacetate (FDA, green) and propidium iodide (PI, light purple). Z-stack images were captured using CLSM and then reconstructed. Arrowheads indicate a dead root hair cell line (light purple). **(F)** Enlarged images of root epidermis stained with FDA and PI.

*fugu5-1 ppa1 ppa5* triple mutants showed normal fertility, but their phenotype was more severely affected than that of *fugu5-1 ppa1*, even on MS plates (Figures 9A,B). The cotyledons and true leaves of *fugu5-1 ppa1 ppa5* developed without adhesion or strong atrophy, which was also observed in the triple mutants *fugu5-1 ppa1 ppa2*, *fugu5-1 ppa1 ppa4*, and *fugu5-3 ppa2 ppa4*, suggesting negligible contribution of PPa5 to the development of cotyledons and leaves (Figures 9A,C). In contrast to shoots, the roots of *fugu5 ppa1 ppa5* were significantly shortened and had fewer root hairs (Figures 9A,B). The PPa5-GFP signal was observed only in root epidermis cells, and was particularly strong in root hair cells (Figure 9D). Staining of roots with FDA and PI, which visualized live/dead cells, indicated death of root epidermal cells in the root hair lines of *fugu5-1 ppa1 ppa5* (Figures 9E,F). Furthermore, root cells of the triple mutant were markedly shortened, even in the mature region, compared with those of *fugu5-1 ppa1* (Figure 9E). These observations suggest that PPa5 functions as active PPase in the mature root epidermis, mainly in root hair line cells where it acts in combination with H^+^-PPase and PPa1. By combining the phenotypes of triple mutants described above, the physiological importance of individual sPPase in various tissues could be determined, and is summarized in Table 3.

**TABLE 3:** Tissue-specific phenotypes of single and multiple mutants of H^+^-PPase and sPPases.

**Organ**	**Mutant line**	**References**
**Leaf**		
Decreased size	*fugu5-3*	Asaoka et al. (2016)
Stomatal closure delay	*fugu5s*	Asaoka et al. (2019)
Altered pavement cell	*fugu5-1*	This study
morphology		
Leaf severe deformity	*fugu5-1 ppa1 ppa2*	This study
	*fugu5-1 ppa1 ppa4*	This study
Leaf edge atrophy (strong)	*fugu5-1 ppa1^+/–^ ppa4*	This study
Leaf edge atrophy (mild)	*fugu5-1 ppa4*	Segami et al. (2018)
Leaf damage (mild)	*fugu5-1 ppa1*	Segami et al. (2018)
**Root**		
Root tip swelling (Severe)	*fugu5-1 ppa1 ppa5*	This study
(Strong)	*fugu5-1 ppa1*	Segami et al. (2018)
(Strong)	*fugu5-1 ppa1^+/–^ ppa2*	This study
(Mild)	*fugu5-1 ppa2*	Segami et al. (2018)
Root epidermal cell death	*fugu5-1 ppa1 ppa5*	This study
**Cotyledon**		
Impaired cotyledon	*fugu5-1 ppa1 ppa2*	This study
	*fugu5-1 ppa1 ppa4*	This study
	*fugu5-3 ppa2 ppa4*	This study
Altered pavement cell morphology and microtubule dynamics	*fugu5-1, fugu5-3*	Gunji et al. (2020)
Elliptical shape	*fugu5s*, *vhp1-1*	Ferjani et al. (2011)
**Hypocotyl**		
Short hypocotyl (strong)	*fugu5-1 ppa1*	Segami et al. (2018)
Short hypocotyl (mild)	*fugu5-1*, *fugu5-3*, *vhp1-1*	Ferjani et al. (2011)
**Flower**		
Sterile pistil	*fugu5-3 ppa2 ppa4*	This study
Jaggy petal	*fugu5-3 ppa2 ppa4*	This study
**Embryo, seed**		
Nearly lethal	*fugu5-1 ppa1 ppa2*	This study
	*fugu5-1 ppa1 ppa4*	This study
Enlargement (strong)	*fugu5-1 ppa1^+/–^ ppa2*	This study
	*fugu5-1 ppa1^+/–^ ppa4*	This study
Enlargement (mild)	*ppa1 ppa2 ppa4 ppa5*	This study
	*fugu5-1*	This study

## Discussion

### Physiological Mechanism of Leaf Atrophy

Leaf atrophy was observed in *fugu5* mutants when grown on ammonium-free medium. This phenotype has been shown to be triggered by excessive PPi, and thus could be rescued through heterologous expression of the yeast sPPase IPP1 in *fugu5* mutants (Fukuda et al., 2016). In this study, we focused on two questions: why the atrophic phenotype depends on direct contact of shoots with medium, and the mechanism through which ammonium ions prevent leaf atrophy. To address the first question, we analyzed mutant leaves and observed several new phenotypes: irregular and disconnected leaf veins (Figure 1), abnormal mesophyll tissue organization, cell swelling and severely simplified pavement cells (Figure 2), decrement of β-glucans (Figure 3), and partial lack of cuticle layer in the rosette leaves (Figure 4). These observations led us to consider dysfunctional biosynthesis of cell wall components and cutin. During biosynthesis of these macromolecules, PPi is generated (Heinonen, 2001). The increased level of PPi may suppress biosynthesis of these macromolecules and thus cause defects in cell wall construction and strength in growing leaves.

In the case of disconnected veins (Figure 1), a similar phenotype was reported in a triple mutant of glycosyltransferases in the cellulose synthase-like D (CSLD) family, *csld2 csld3 csld5*, which transfer mannose from GDP-mannose onto an endogenous acceptor (Yin et al., 2011). GDP-mannose is a nucleotide-sugar that releases PPi from its synthesis reaction, and PPi accumulation likely inhibits this reaction, as recently reported for UDP-glucose (Ferjani et al., 2018). Indeed, the etiolated hypocotyl of *fugu5-1 ppa1* contained a reduced level of mannose (Segami et al., 2018). Along with the decreased level of β-glucans in the leaf surface (Figure 3), cell wall synthesis was markedly inhibited in *fugu5-1* leaves grown on ammonium-free medium.

The cuticle, which acts as plant skin, is essential for the protection of leaves and structural support of tissues. Therefore, the lack of cuticle layer in *fugu5* grown on MGRL medium may result in serious damage to its tissues. In the early pathway of cuticle synthesis, ligation of CoA with long chain fatty acid by acyl-CoA synthetase (EC6.2.1.3) releases PPi into the cytosol (Pulsifer et al., 2012; Fich et al., 2016). This finding strongly suggests that the lack of VHP1 increases the PPi level in leaf epidermal cells and suppresses the formation of cuticle. Cells with immature cell walls (Figure 3) lacking the cuticle layer (Figure 4) might be physically weak and sensitive to osmotic fluctuations in the extracellular environment. Taken together, these findings indicate that leaf atrophy in *fugu5* might be caused by cell death due to external stresses, such as infiltration of solutes from the growth medium.

### The Importance of H^+^-PPase and sPPases in Leaf Development

To address the second question regarding the physiological mechanism through which ammonium ion prevents the atrophy in *fugu5* and other mutants, we hypothesized three possible effects of ammonium in the mutants: induction of sPPases, induction of other pyrophosphatases, and suppression of PPi generation. The first hypothesis was clearly contradicted by the results of immunoblotting (Figure 6) and quantitative real-time polymerase chain reaction (RT-qPCR) (Supplementary Figure 3). We investigated the second possibility based on RT-qPCR and the lethality of multiple knockout mutants, which provides insight into the shared system of several PPases. Accumulation of PPi in cells is known to cause critical growth arrest and cell death through inhibition of fundamental metabolic reactions involving DNA and NAD (Chen et al., 1990; Serrano-Bueno et al., 2013). If ammonium induced other PPi remover(s), multiple knockout mutants of H^+^-PPase and sPPases would survive on ammonium-containing medium.

We constructed four triple mutants, *fugu5-1 ppa1 ppa2*, *fugu5-1 ppa1 ppa4*, *fugu5-1 ppa1 ppa5*, and *fugu5-3 ppa2 ppa4*, and tested their growth on half-strength MS plates, which contained 10 mM NH_4_^+^. *fugu5-1 ppa1 ppa2* and *fugu5-1 ppa1 ppa4* showed the severest phenotypic effects, which were nearly lethal (Tables 1, 2 and Figure 8). Among their heterozygous counterparts, *fugu5-1 ppa1^+/–^ ppa4* showed significant leaf edge atrophy (Figures 8A,B), whereas *fugu5-1 ppa1^+/–^ ppa2* showed no atrophic leaf symptoms (Supplementary Figure 6A). These results indicate that PPa4 is more important than PPa2 for preventing leaf atrophy (Supplementary Figures 6B,C). The other triple mutant, *fugu5-3 ppa2 ppa4*, showed no additional effects on leaf phenotype compared to the former two mutants (Supplementary Figure 7). Comparison of *fugu5-3 ppa2 ppa4* with *fugu5-1 ppa1 ppa2* and *fugu5-1 ppa1 ppa4* clearly indicates the importance of PPa1 to leaf development. In GFP localization analysis for our previous study (Segami et al., 2018), VHP1-GFP and PPa1-GFP were expressed in all leaf cell types, whereas PPa2-GFP and PPa4-GFP were preferentially expressed in mesophyll and epidermis cells, respectively. Therefore, the severe phenotype of *fugu5-1 ppa1 ppa2* (Figure 8D) may be related to mesophyll dysfunction, whereas *fugu5-1 ppa1 ppa4* (Figure 8E) suffers from defects in epidermal development. PPa5-GFP is also expressed in leaf epidermis, but is limited to mature leaves, whereas PPa4-GFP is highly expressed during the early stages of leaf development. Combined with the result that *fugu5-1 ppa1 ppa5* and *fugu5 ppa5* did not exhibit strong leaf atrophy (Figures 5A, 9C), this shows that the atrophic phenotype is associated with young leaves, in agreement with the notion that PPi is abundantly released in proliferating young tissues. Therefore, the combination of VHP1, PPa1, PPa2, and PPa4 might be essential for proper leaf development. The present observations of nearly lethal phenotypic effects in multiple mutants of VHP1 and PPases strongly suggest that other PPi utilizing enzymes or PPi hydrolyzing enzymes, if any exist, are not involved in PPi homeostasis.

### Phenotypes of Embryos and Root Hairs in Triple Mutants

The triple mutants *fugu5-1 ppa1 ppa2* and *fugu5-1 ppa1 ppa4* both showed nearly lethal phenotypes. Their embryos exhibited retarded growth and developed without showing the typical heart-stage and torpedo-stage shapes, becoming round in shape at 8 DAP. Finally, *fugu5-1 ppa1 ppa4* made green embryos lacking differentiation into cotyledons and hypocotyls in seeds at 14 DAP (Figure 7A and Supplementary Figure 5). In this study, we could not obtain triple homozygous mutants from seeds stored that were over 1 year (Tables 1, 2). In combination with the observation that *fugu5-1 ppa1 ppa4* embryos remained green in color until 14 DAP (Figure 7A and Supplementary Figure 5), this result suggests that seed maturation in the mutant is insufficient for maintaining seed longevity during long-term storage. The high ratio of shrunken seeds to normal-sized ones (Figure 7B) and low germination rate suggest that PPa2 is more important than PPa4 during embryogenesis.

*fugu5-1 ppa1 ppa5* showed phenotypes of short roots, short root cells, fewer root hairs, and necrosis of the root epidermis (Figures 9A,B,E,F). Root epidermis is divided into two types: root hair cells and non-root hair cells (Galway et al., 1994; Berger et al., 1998). Notably, root epidermis is normally viable at an early developmental stage, before root-hair formation (Figure 9E). PPa5-GFP was expressed primarily in root hairs in the root elongation zone (Figure 9D) and PPa3-GFP was also expressed in root hair cells, whereas PPa4-GFP was mainly detected in non-root hair cells and PPa2-GFP was expressed only during the early developmental stage (Segami et al., 2018). These observations coincide with the defective root hair phenotype of *fugu5-1 ppa1 ppa5*, in which root hair line cells died (Figure 9E). In this triple mutant, most root hairs died, but several elongated hairs were observed (Figures 9A,E), suggesting that the formation and elongation of root hairs require precise regulation of PPi at a critical level and the remaining PPa3 activity was insufficient to maintain viability.

### Possible Mechanism for Rescue of Mutant Phenotype by Ammonium

To explore the relationship between PPi production and absence of ammonium supply, here we considered organ-specific nitrogen metabolism. Most plants incorporate and utilize ammonium preferentially over nitrate (Howitt and Udvardi, 2000; Konishi et al., 2017). Thus, *A. thaliana* incorporates NH_4_^+^ preferentially from MGRL^Am^ medium and converts NH_4_^+^ to amino acids in roots to reduce the toxic NH_4_^+^ levels (Hachiya and Sakakibara, 2017). The amino acids thus generated are transported from the roots to shoots (Konishi et al., 2017). In contrast, NO_3_^–^ is incorporated by roots, and then transported directly from roots to shoots because NO_3_^–^ has no toxicity to plant cells. In shoots, NO_3_^–^ is reduced to NH_4_^+^ via NAD(P)H reducing equivalents that are mainly supplied by photosynthesis and is subsequently incorporated into glutamine. The assimilation of nitrate is assumed to act as a strong consumer of reducing power, and therefore ammonium utilization greatly decreases the energy consumption required to synthesize organic N compounds (Williams et al., 1987; Hachiya and Sakakibara, 2017; Gakière et al., 2018).

Considering the differences in nitrogen metabolism between roots and shoots, metabolism of NO_3_^–^ into amino acids occurs preferentially in shoots grown on MGRL medium. Although NO_3_^–^ reduction itself includes no PPi-generating reactions, for example, the biosynthesis of NAD, which is involved in NO_3_^–^ reduction, generates PPi (Heinonen, 2001; Gakière et al., 2018). We predict that the elevated level of PPi formed from NO_3_^–^ reduction related pathway in shoots causes cellular dysfunction and atrophy in *fugu5* plants grown on MGRL medium. The addition of ammonium to the medium might reduce the generation of PPi in shoots by promoting amino acid synthesis in roots. This organ-specific nitrogen metabolism for synthesis of amino acids rescues the phenotype of *fugu5*. To further test this hypothesis, we will analyze transcriptomic and metabolomic data and then identify the pathways generating high PPi levels under ammonium-free conditions.

## Data Availability Statement

All datasets generated for this study are included in the article/Supplementary Material.

## Author Contributions

MF co-coordinated the project, contributed to phenotyping, analyzed the data, and drafted the manuscript. MMi conducted microscopy and calcofluor white staining. MMa conceived of and initiated the project, obtained funding, and contributed to the manuscript. SS conducted association analysis, constructed multiple mutants, wrote and finalized the manuscript. AF provided the *fugu5* mutants, *AVP1_pro_::IPP1* transgenic lines and contributed to the manuscript. SK conducted image analysis. RS conducted RT-qPCR analysis. TT contributed to seed phenotype analysis. All authors read and approved the final manuscript.

## Conflict of Interest

The authors declare that the research was conducted in the absence of any commercial or financial relationships that could be construed as a potential conflict of interest.

## Abbreviations

DAG: days after germination
DAP: days after pollination
H^+^-PPase: H^+^-translocating pyrophosphatase
PFP: PPi-dependent phosphofructokinase
PPDK: pyruvate orthophosphate dikinase
PPi: inorganic pyrophosphate
sPPase: soluble inorganic pyrophosphatase
UGPase: UDP-glucose pyrophosphorylase.
