# Supplementary material for: Lack of Vacuolar H+ -Pyrophosphatase and Cytosolic Pyrophosphatases Causes Fatal Developmental Defects in Arabidopsis thaliana
Source: Front Plant Sci. 2020 May 26;11:655. doi: 10.3389/fpls.2020.00655 (PMC7266078; doi:10.3389/fpls.2020.00655)
Supplement: Supplementary file 1 [file Data_Sheet_1.docx]

**Supplemental Methods**

**Quantitative Real-Time PCR**

Whole plants of 10- and 20-day-old seedlings were frozen in liquid nitrogen and powdered with a mortar and pestle. RNA was extracted from 100 mg of each frozen powdered sample using the RNAeasy a Plant Mini Kit (Qiagen, Valencia, CA, USA) and considered the total RNA fraction. First-strand cDNA was synthesized from 1.0 µg of total RNA in a 20-µL reaction volume (200 ng/mL) via reverse transcription using the iScript cDNA Synthesis Kit (Bio-Rad Laboratories). Quantitative PCR (qPCR) was conducted on a Thermal Cycler Dice real-time system (Takara Bio, Otsu, Japan) using SYBR Premix Ex Taq (Takara Bio). Primer sets used for PCR to detect the genes are listed in Supplemental Table 1. Quantification was performed three times for each line. The PCR conditions were as follows: one cycle at 95°C for 1 min followed by 40 cycles with denaturation at 95°C for 15 s and annealing at 60°C for 45 s. The conditions of the melt curve reaction followed recommendations for the instrument and reagents.

The mRNA contents of the WT and mutant plants were normalized to the mRNA level of *UBIQUITIN5* (At3G62250) as determined through parallel amplifications, and are shown as relative quantities to that of *UBIQUITIN5*. The primer sets used are listed in Supplemental Table 1. Previously published sequences for *PPa1*, *PPa2*, and *PPa5* (Öztürk et al., 2014) and *UBIQUITIN5* (Che et al., 2002) were used for primer design.

**Extraction and Measurement of Nitrate in Plants Shoots or Leaves**

Extraction and measurements were performed according to the method of Takatani et al. (2014). Whole shoot (10-DAG) and one leaf (20-DAG) were used for the extraction. Nitrate in shoot or in a leaf was measured using the Flow Injection Analyzer (PFA-310NO, FIA Co).

**Extraction and Measurement of Ammonium in Plant Shoots**

Extraction and measurements were performed according to the method of Hachiya et al. (2017). 10-DAG and 20-DAG whole shoots were used for extraction. Measurement of ammonium was conducted according to the instructions of Ammonia Test Wako (Wako Pure Chemical Industries). The amount of ammonium in shoot was calculated by the following formula.

NH_4_^+^ (μmol / g FW) = NH_4_^+^ (μM) × extracted volume (mL) / shoot g FW / 1000

**Supplemental Table 1 ⎢ Primer sets used for PCR to detect the genes encoding PPa1, PPa2, PPa4, PPa5, and PFP subunits.**

|  |  | Forward | Reverse |
| --- | --- | --- | --- |
| PPa1 | At1G01050 | 5'-ACAATCGGCTGTTTCGTTTC-3' | 5'-TTCCTTTAGTGATCTCAACAACCAC-3' |
| PPa2 | At2G18230 | 5'-GATTCTCTGCTTCGGTTTCG-3' | 5'-CAGTAGGAGCTTCTGGACCAATC-3' |
| PPa4 | At3G53620 | 5'-ATCCGCCGTTTCTTTGAAG-3' | 5'-AATGCTGAACTGCGTCGTAG-3' |
| PPa5 | At4G01480 | 5'-CTCCACACTTTCCGCAAGAT-3' | 5'-ACTGGAGCTCCAGGTCCG-3' |
| PFP β subunit | At1G12000 | 5'-GACATGATCTGCAGTGGAAGAGAC-3' | 5'-GCATTGGTGTTGGAATCATCTCC-3' |
| PFP β subunit | At4G04040 | 5'-GGAAATGTTATGATCGATGCGCG-3' | 5'-GTAATGTGAGAAGCAGCACGACCC-3' |
| PFP α subunit | At1G76550 | 5'-CTGGAGATCACTGATGAGGTCCTC-3' | 5'-CAGCATTGACCTGTTCGGTTGTTC-3' |
| PFP α subunit | At1G20950 | 5'-GCTCGGAAGAACTAAGGATCAGATC-3' | 5'-GCTGCATCTGTGTTTGATATTACGC-3' |
| Ubiquitin5 | At3G62250 | 5'-CTTGAAGACGGCCGTACCCTC-3' | 5'-CGCTGAACCTTTCAAGATCCATCG-3' |


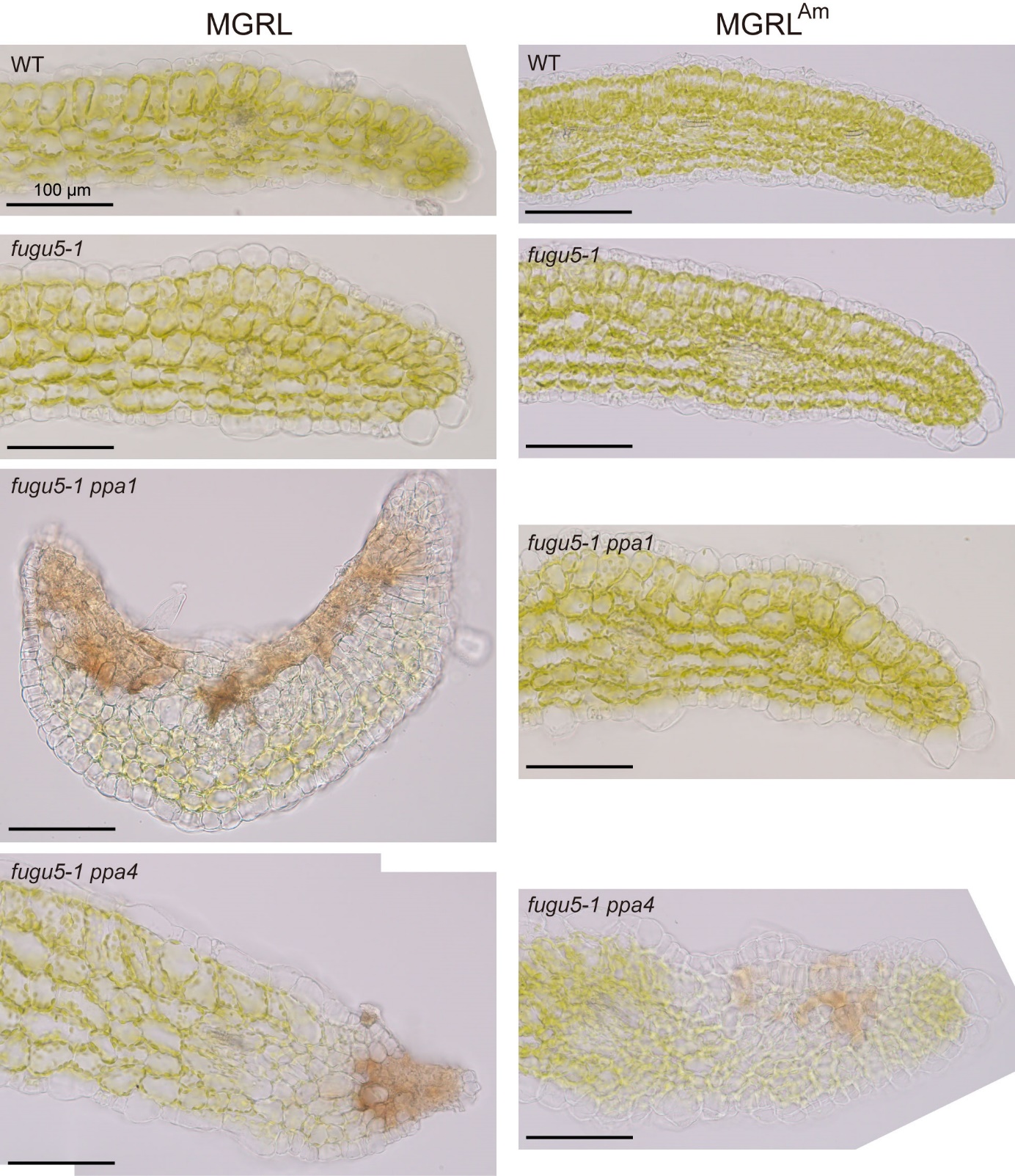


**Supplemental Figure 1 ⎢Cross section of the first leaves of double mutants.** First leaf cross sections of 10-DAG (WT and *fugu5-1*) or 12-DAG plants (*fugu5-1 ppa1* and *fugu5-1 ppa4*) at the center of the leaf. Light brown color in the double mutants suggests cell death.


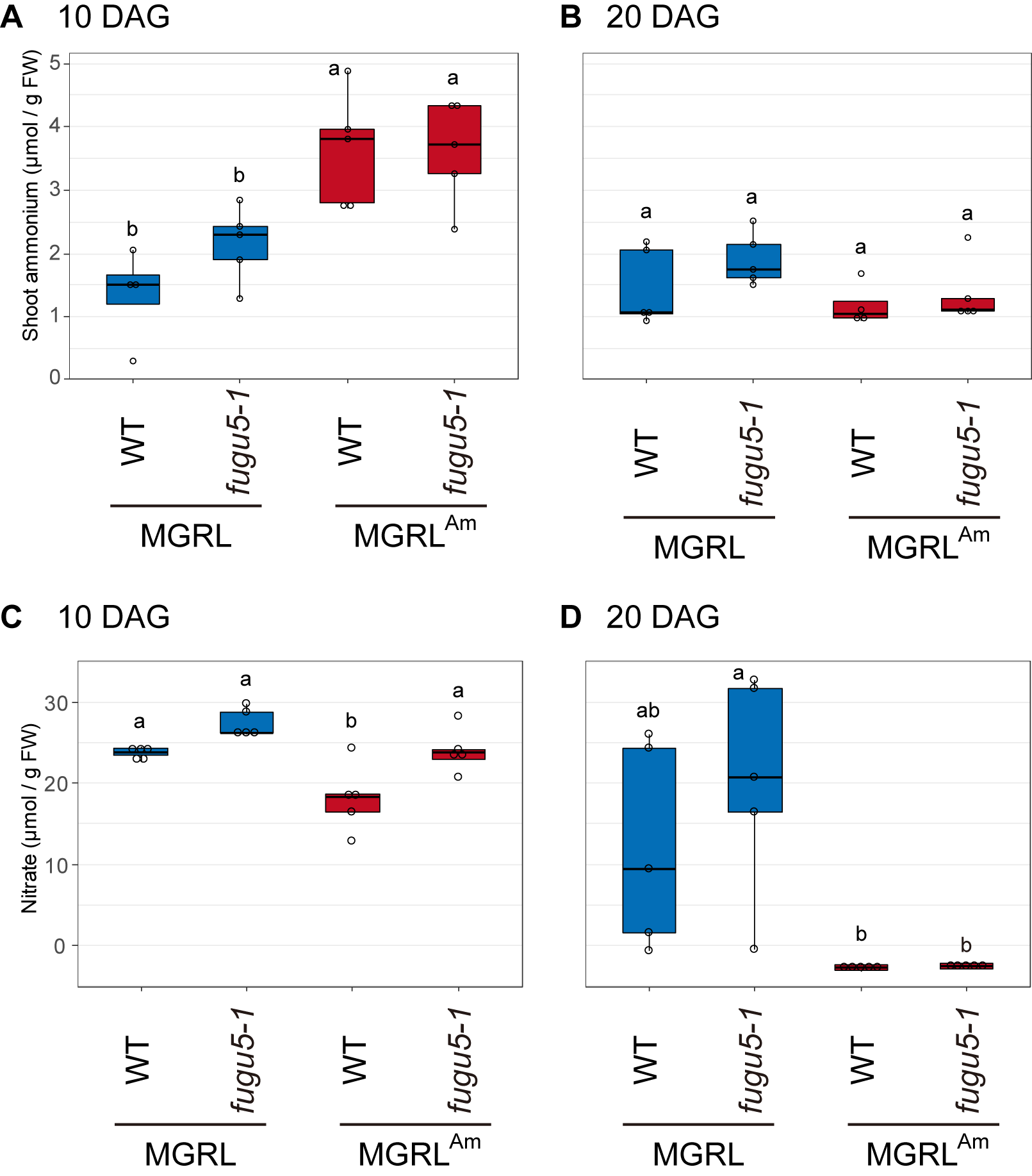


**Supplemental Figure 2 ⎢ Measurement of ammonium and nitrate in pant shoots**

The amount of ammonium and nitrate in shoots grown on MGRL or MGRL^Am^ medium were measured. Different letters above each bar indicate statistically significant differences (*P* < 0.05, Tukey’s HSD test).


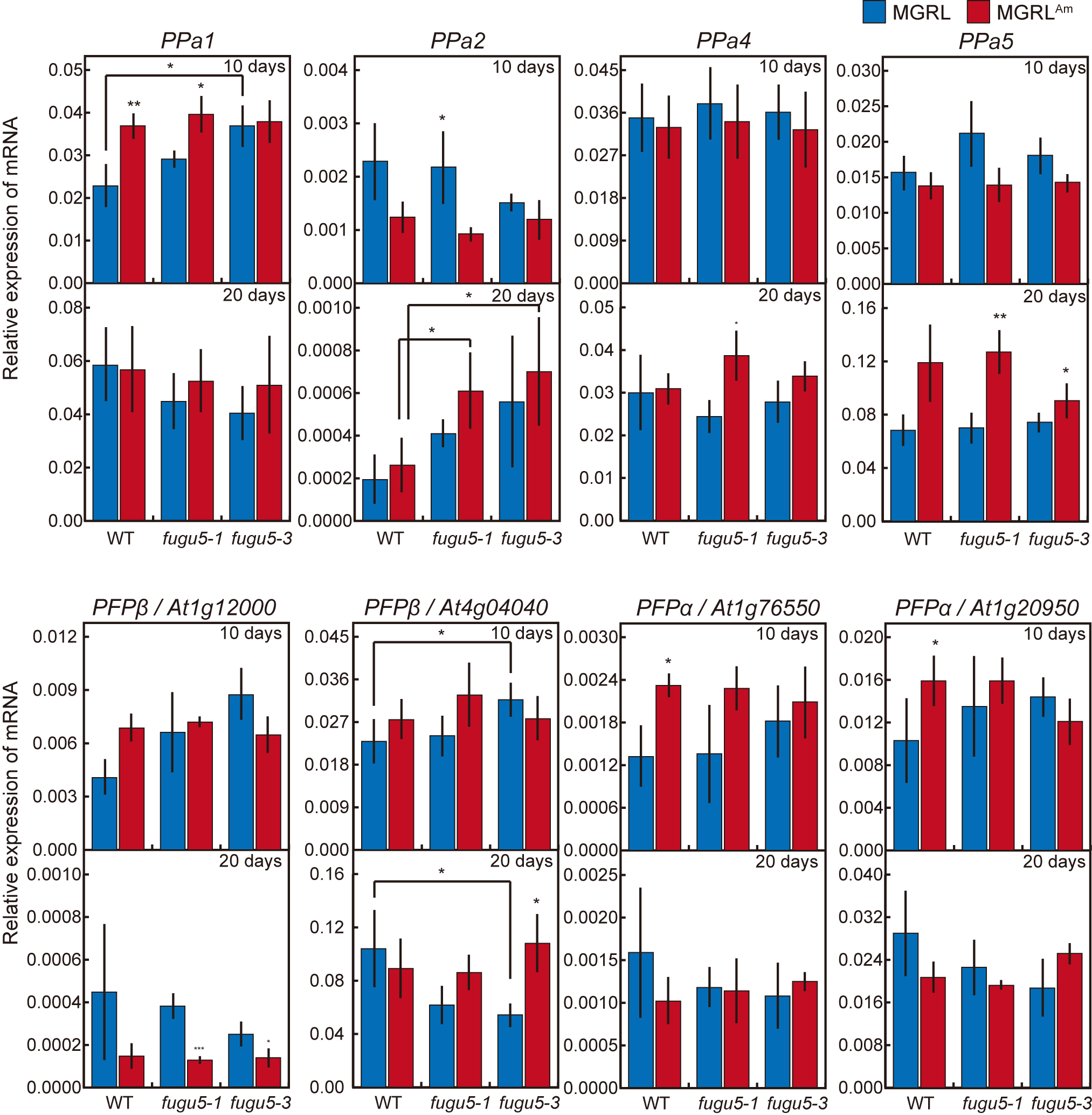


**Supplemental Figure 3 | Expression levels of sPPase and PFP in *fugu5-1.*** Transcript levels of sPPases (*PPa1*, *PPa2*, *PPa4*, *PPa5*) and PPi-dependent phosphofructokinase, which hydrolyzes PPi in the cytosol. mRNA levels were measured using whole 10- and 20-day-old plants. Lower panels are of the subunits of PPi-dependent phosphofructokinase (PFP): At1G12000 (PFP β subunit), At4G04040 (PFP β subunit), At1G76550 (PFP α subunit), and At1G20950 (PFP α subunit). Transcript levels are normalized to the mRNA level of ubiquitin5 (At3G62250). Asterisks indicate significant differences at **P* < 0.05, ***P* < 0.01, ****P* < 0.005 (Student’s *t*-test, n = 4, Error bars = standard deviation).

**
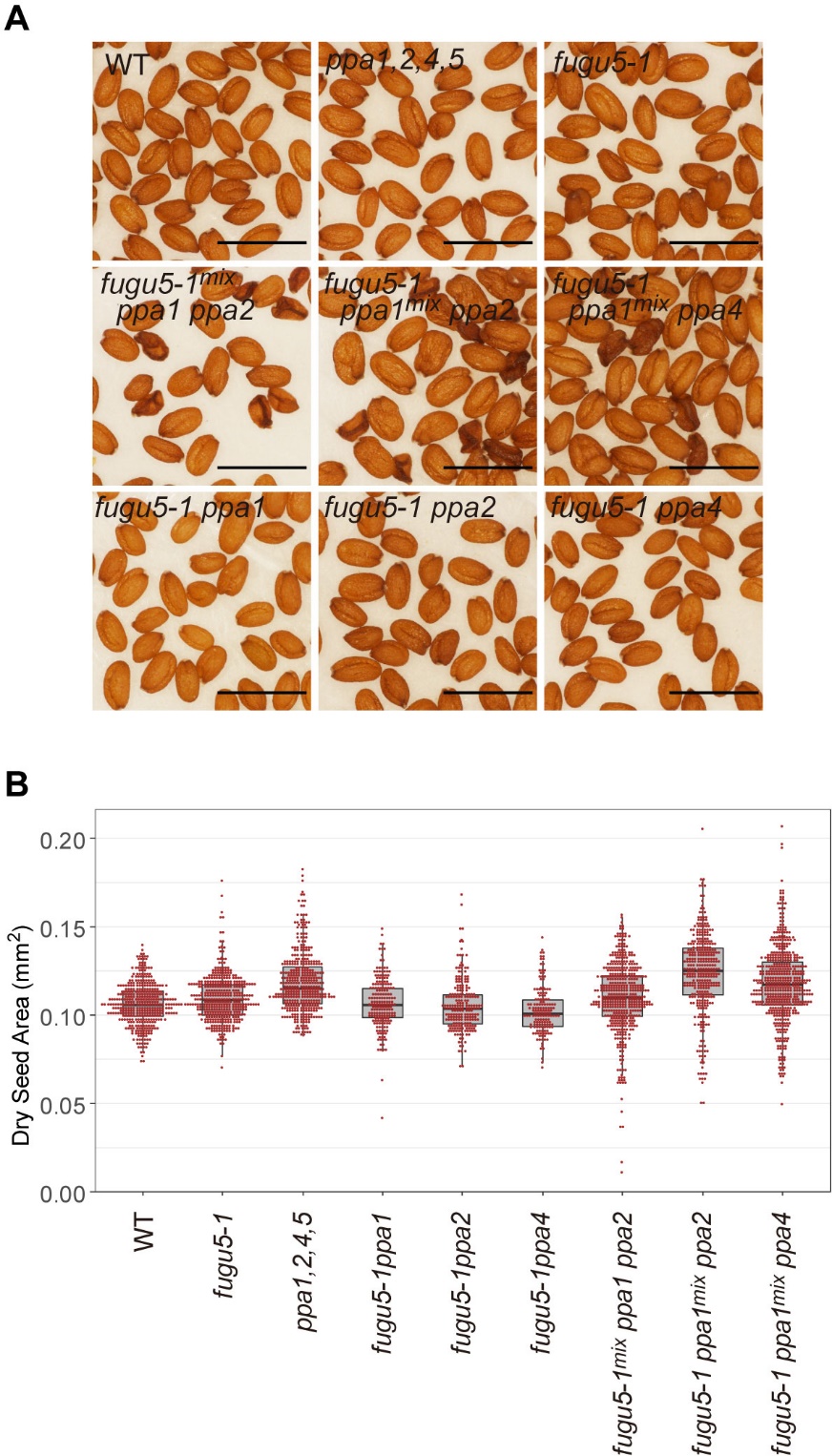
**

**Supplemental Figure 4 ⎢ Phenotypes of mature seeds of single, double, and triple mutants.**

**(A)** Stereomicroscopy of dry seeds. **(B)** Dry seed size quantified by area (mm^2^). **(C)** Imbibed seed size quantified by area (mm^2^). Sown seeds on plates were imaged with an upright microscope. For quantification of seed size, images were segmented using Color Thresholder and then measured with the analyze particle command in Image J. For details, see Supplemental Figure 8.

**
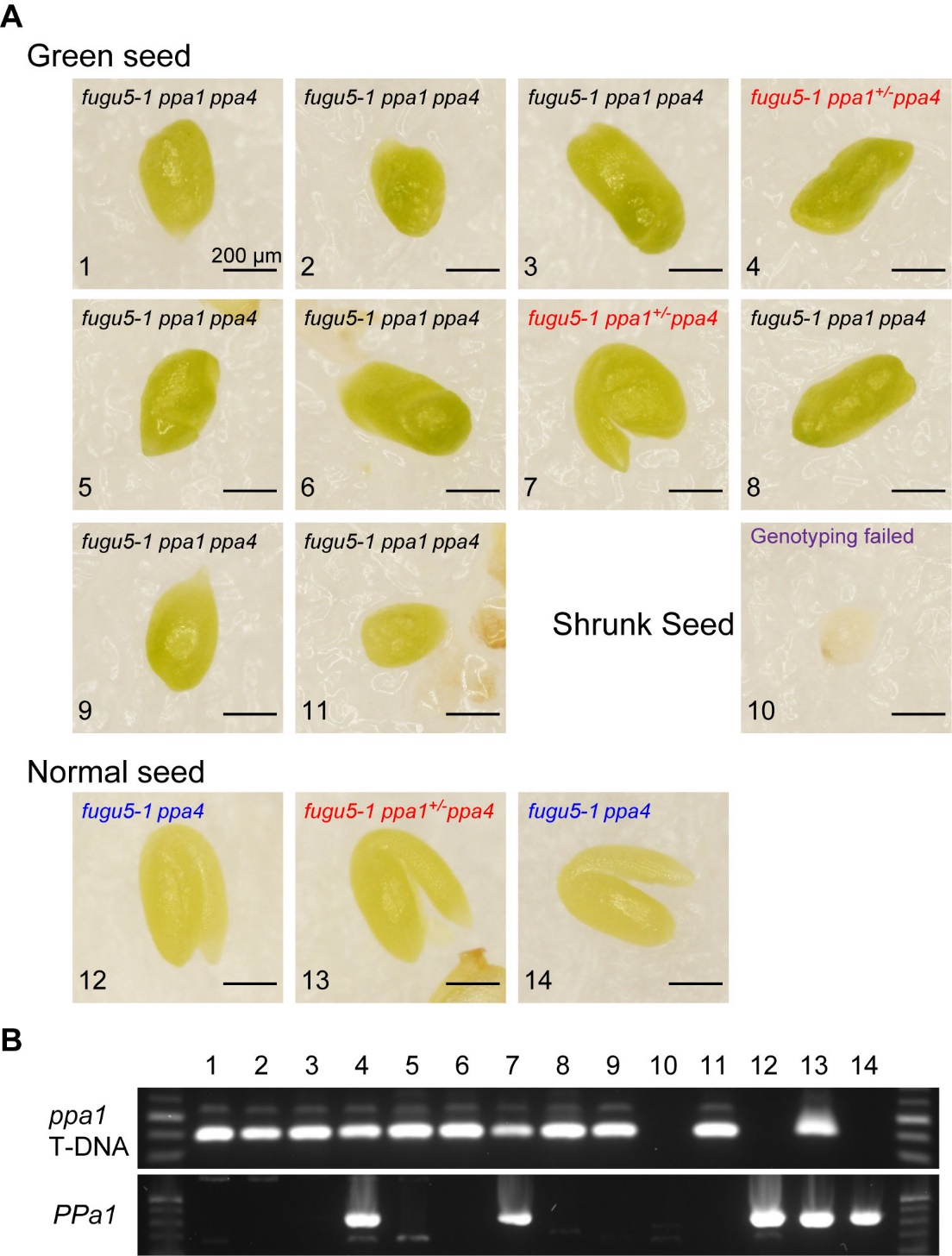
**

**Supplemental Figure 5 ⎢Genotyping of the *fugu5-1 ppa1^+/–^ ppa4* green embryo.**

**(A)** Embryos isolated from *fugu5-1 ppa1^+/–^ ppa4* 14-DAP mature fruits observed using a stereomicroscope. The embryos were classified into normal, green, and shrunken seeds based on their morphological properties. **(B)** Genotypes of the embryos shown in panel (A). Numbers correspond to those in (A). Labels on the left side, *ppa1* T-DNA and *PPa1*, indicate T-DNA-specific amplification and the endogenous sequence without T-DNA amplification, respectively.

**
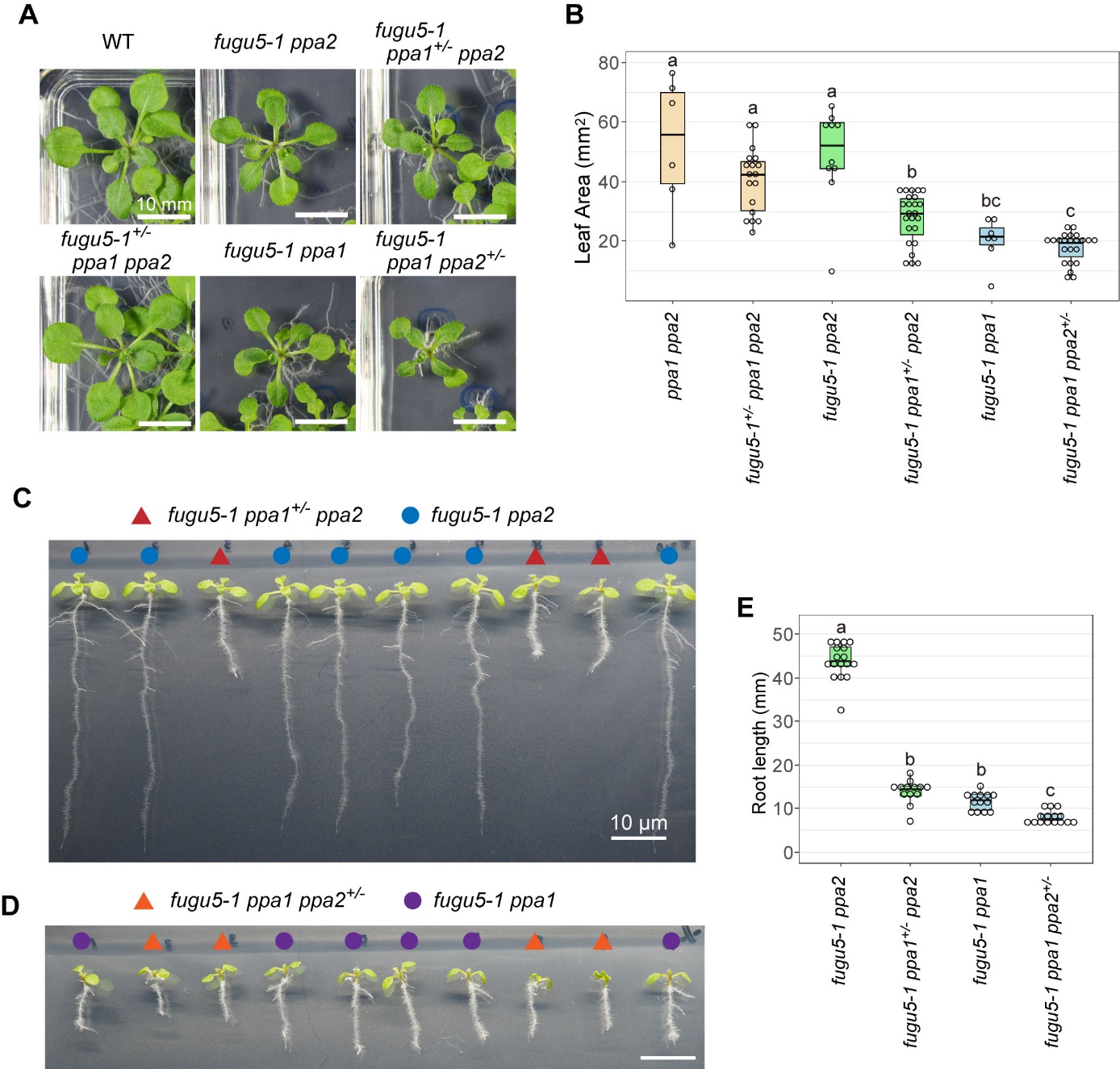
**

**Supplemental Figure 6 ⎢ Growth phenotype of *fugu5-1 ppa1 ppa2* heterozygous plants.** WT, *fugu5-1 ppa2*, *fugu5-1 ppa1*, *fugu5-1 ppa1*^+/–^ *pa2*, *fugu5-1*^+/–^ *ppa1 ppa2*, and *fugu5-1 ppa1 ppa2*^+/–^ were grown on half-strength MS plates. **(A)** Leaves of WT and mutants. **(B)** Comparison of leaf area showing the growth of seedlings. **(C)** Roots of 9-DAG *fugu5-1 ppa1^+/–^ ppa2* and *fugu5-1 ppa2* cultivated on a vertical plate. **(D)** Roots of 9-DAG *fugu5-1 ppa1 ppa2*^+/–^ and *fugu5-1 ppa1*. **(E)** Comparison of root length. Different letters above each bar indicate statistically significant differences (*P* < 0.05, Tukey’s HSD test).

**
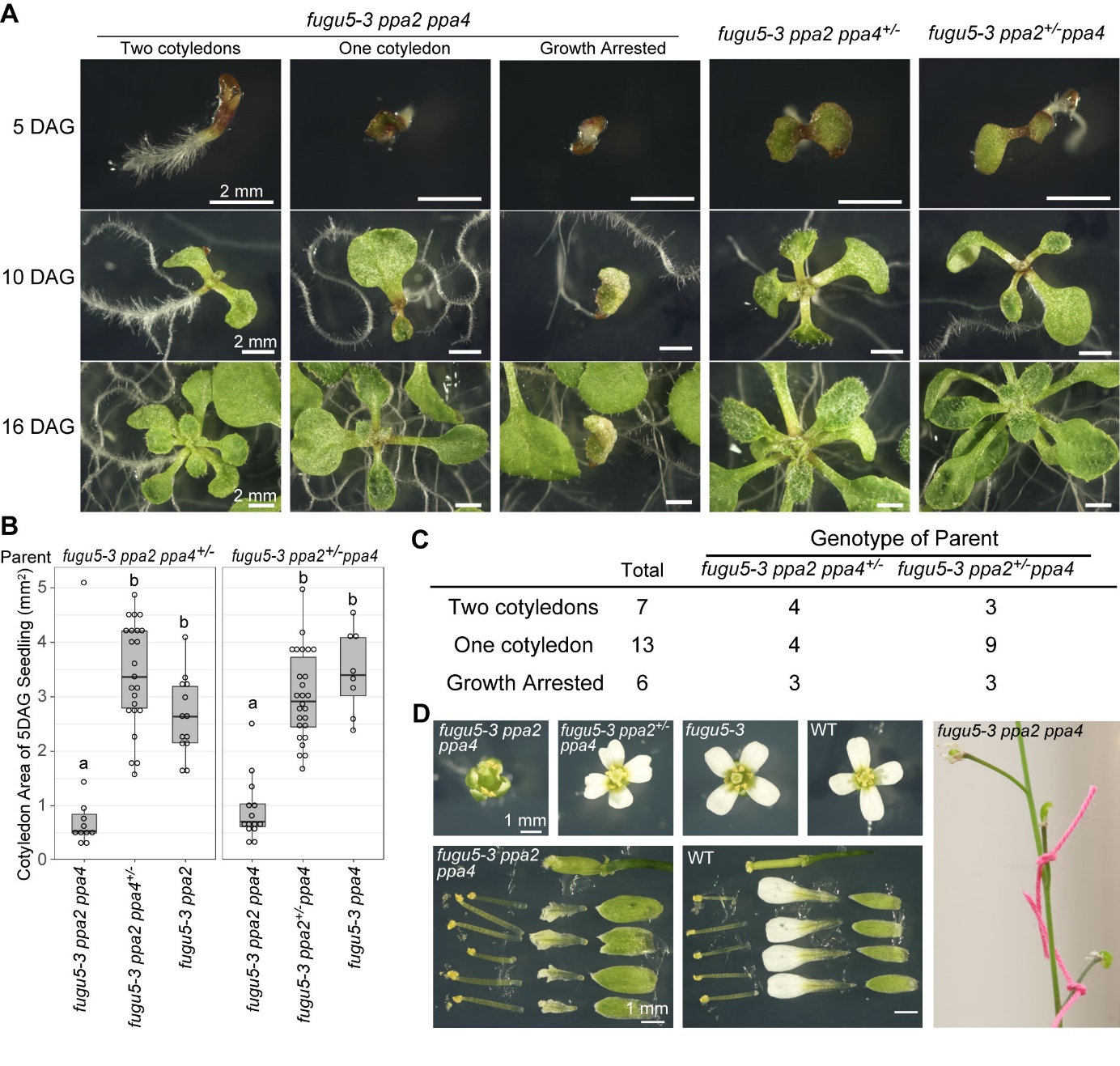
**

**Supplemental Figure 7 ⎢ The triple mutant *fugu5-3* *ppa2 ppa4* shows severe germination defects. (A)** Growth of *fugu5-3 ppa2 ppa4* homozygous (three panels on left) or heterozygous (two panels on right) 5-, 10-, and 16-DAG seedlings. Typical seedlings with two cotyledons (normal), a single cotyledon, and with germination defects are shown. **(B)** Comparison of cotyledon size in 5-DAG seedlings of *fugu5-3 ppa2 ppa4* heterozygous. Different letters above each bar indicate statistically significant differences (*P* < 0.01, Steel-Dwass test). **(C)** Classification of germinated seeds by cotyledon shape and development of true leaves. **(D)** Floral organ morphology of *fugu5-3 ppa2 ppa4*. Pink strings mark fruits of the mutant that had been hand pollinated using WT pollen.

**Supplemental Figure 8 ⎢Image J / FIJI Macro for area quantification**

size = 0.05;//Lower limit of "Analyze particle"

results = true;//Clear results before analysis

jpg = 80;//Quality of jpeg

//parameters for scale setting

distance = 331;

known = 10;

unit = "mm";

dir = getDirectory("image");

title = getTitle();

dotIndex = indexOf(title, ".");

name = substring(title, 0, dotIndex);

format = substring(title, dotIndex);

if(format == ".JPG") format2 = ".jpg";

else format2 = format;

run("Set Measurements...",

"area mean shape limit display add " +

"decimal=3");

run("Overlay Options...", "stroke=cyan width=5 fill=none set");

if(results == true) run("Clear Results");

if(roiManager("count") != 0) roiManager("reset");

setBatchMode(true);

run("Duplicate...", "title=[" + name + "_Binary1" + format + "]");

// Color Thresholder 2.0.0-rc-69/1.52p

// Autogenerated macro, single images only!

***Paste “Color Thresholder” script here***

// Colour Thresholding-------------

save(dir + name + "_Binary1" + format2);

close();

setBatchMode(false);

open(dir + name + "_Binary1" + format2);

title0 = getTitle();

dir = getDirectory("image");

selectImage(1);

run("Select None");

title1 = getTitle();

dotIndex1 = lastIndexOf(title1, ".");

name1 = substring(title1, 0, dotIndex1);

format1 = substring(title1, dotIndex1);

selectImage(2);

run("Select None");

title2 = getTitle();

dotIndex2 = lastIndexOf(title2, ".");

name2 = substring(title2, 0, dotIndex2);

format2 = substring(title2, dotIndex2);

selectWindow(title2);

run("Set Scale...", "distance=distance known=known pixel=1 unit=unit");

setFont("SansSerif", 18);

setForegroundColor(150, 150, 150);

drawString(distance + "pixel =" + known + unit, 5, 20);

run("Set Measurements...",

"area mean shape limit display add " +

"redirect=None decimal=3");

setThreshold(200, 255);

run("Analyze Particles...", "size=[" + size + "]-infinity show=Overlay display exclude add");

save(dir + name2 + "_R" + format1);

saveAs("Results" , dir + name1 + "_Results.csv" );

roiManager("Save", dir + name1 + "_" + distance + "_" + known + unit + ".zip");

selectWindow(title1);

roiManager("Show All");

run("Tile");

**References**

Che, P., Gingerich, D.J., Lall, S., and Howell., S.H. (2002) Global and hormone-induced gene expression changes during shoot development in *Arabidopsis*. *Plant Cell* 14: 2771–2785.

Hachiya. T. and Okamoto, Y. (2017) Simple spectroscopic determination of nitrate, nitrite, and ammonium in *Arabidopsis thaliana*. *Bio-protocol*, 7 (10), e2280.

Öztürk, Z.N., Greiner, S., and Rausch, T. (2014) Subcellular localization and developmental regulation of cytosolic, soluble pyrophosphatase isoforms in *Arabidopsis thaliana*. *Turk J. Bot.* 38: 1036–1049.

Takatani, N., Ito, T., Kiba, T., Mori, M., Miyamoto, T., Maeda, S. and Omata, T. (2014) Effects of high CO_2_ on growth and metabolism of *Arabidopsis* seedlings during growth with a constantly limited supply of nitrogen. *Plant Cell Physiol.*, 55, 281–92.
